# Acknowledgement to Reviewers of *Cancers* in 2019

**DOI:** 10.3390/cancers12010243

**Published:** 2020-01-19

**Authors:** Cancers Editorial Office

**Affiliations:** MDPI, St. Alban-Anlage 66, 4052 Basel, Switzerland

The editorial team greatly appreciates the reviewers who have dedicated their considerable time and expertise to the journal’s rigorous editorial process over the past 12 months, regardless of whether the papers are finally published or not. In 2019, a total of 2096 papers were published in the journal, with a median time to first decision of 15 days and a median time from submission to publication of 38 days. The editors would like to express their sincere gratitude to the following reviewers for their generous contribution in 2019:
Aasen, TrondLodola, AlessioAbad, CatalinaLoewer, AlexanderAbdel-Nasser, MohamedLöffek, StefanieAbdelrazik, HebaLoganzo, FrankAbe, HiroyukiLoges, SonjaAbe, TakanoriLoges, SonjaAberger, FritzLöhr, MatthiasAbeykoon, JithmaLokeshwar, BalakrishnaAbeywardana, TharindumalaLolkema, Martijn P.Abian, OlgaLomberk, GwenAbid, Karim AlexandreLong, WeiwenAbiko, KaoruLong, YichengAbounader, RogerLongo, AntonioAbraham, DietmarLongy, MichelAbraham, Samuel J.K.Loo, Jia MinAccardi-Gheit, RositaLoo, Ruey LengAcedo Nunez, Maria Del PilarLopez, AngelAcharya, MunjalLópez, José I.Achinger-Kawecka, JoannaLopez, Jose JavierAchreja, AbhinavLópez, Susana B BravoAdamczewski, ZbigniewLópez-Camarillo, CésarAdamek, AgnieszkaLópez-Guerrero, José AntonioAdami, Guy R.López-López, ElixabetAdams, BrianLópez-Pintor Muñoz, Rosa MaríaAdamson, David CoryLorenzo, SempereAdeberg, SebastianLosi, LorenaAdekoya, Timothy O.Losurdo, GiuseppeAdhikari, ManishLotti, Lavinia VittoriaAdliene, DianaLou, EmilAdriana, CeciLou, SongAdunyah, Samuel E.Lou, ZhenkunAdvani, SunilLourenço, Gustavo JacobAeddula, Narothama ReddyLoveland, KateAerts, IsabelleLowes, Lori E.Aerts, Joachim G.Lozano, ElisaAfonso, JulietaLu, JingAgarwal, SumitLu, Jyh-FengAggarwal, Bharat B.Lu, ZhenAghi, Manish K.Luc, CabelAgnelli, LucaLuchini, ClaudioAgodi, AntonellaLuchtel, RebeccaAgostini, MarcoLucia, AlejandroAgresti, AlessandraLuddy, Kimberly A.Ahern, Gerard P.Ludlow, Andrew T.Ahmad, AmirLudovico, PaulaAhmad, FahimLudwig, Dale L.Ahmed, HafizLudwig, MichaelAhmed, Shafiq ULuedde, TomAhn, JaegyoonLui, Dr. Weng-OnnAhn, Joong BaeLui, Vivian Wai YanAidinis, VassilisLuk, Ian Y.Aidinis, VassilisLukong, Kiven EriqueAiolfi, RobertoLum, JulianAkbar, MohammedLund, Elizabeth K.Akbay, EsraLundgren-Åkerlund, EvyAkhavan-Sigari, RezaLundholm, LovisaAkilov, Oleg E.Luni EmdadAkimoto, KazunoriLuo, Chi-WenAkıncılar, Semih CanLuo, MingAkula, ShawLupberger, JoachimAlaibac, MauroLuthra, RajyalakshmiAlaimo, AlessandroLuu, HungAlaimo, AlessandroLuwor, RodneyAlama, AngelaLyng, FionaAlbert, StewartLyons, TraciAldieri, ElisabettaLyu, YuanzhiAl-Dujaili, EmadM’kacher, RadhiaAlentorn, AgustiMa, Dik-LungAleš, RozmanMa, ShuanggeAlesutan, IoanaMa, XingAlexander, H. DenisMaas, MichaelAlexander, MarlieseMaass, KendraAlexandraki, KrystalleniaMaccallini, CristinaAlexandrescu, SandaMaccallini, CristinaAlexiev, Borislav A.Maccaroni, ElenaAlexopoulos, SophoclisMACCHI, AndreaAlgar, ElizabethMacchi, BeatriceAlgül, HannaMacek Jílková, ZuzanaAli, NawabMach, Robert HAli, RameezMachairiotis, NikolaosAli, SimakMachon, OndrejAlifano, MarcoMackenzie, GerardoAljabali, Alaa A AMackenzie, IanAllan, AlisonMacLeod, NicholasAllegra, EugeniaMacNeill, AmyAllegra, SarahMadala, Hanumantha RaoAllegrucci, CinziaMadariaga, AinhoaAllen, Jacqueline E.Maddalena, FrancescaAll-Ericsson, CharlottaMaddirala, Amarendar ReddyAlloza, IraideMadsen, Daniel HargbølAlmeida, RaquelMadureira, Patrícia A.Almeida, Vitor HugoMaeda, ShinAlpaugh, KatherineMaes, KenAlsaab, HashemMaggi, Leonard B.Al-Sawaf, OthmanMaggialetti, NicolaAltieri, FabioMaggiolini, MarcelloAltomare, Deborah A.Magiatis, ProkopiosAltucci, LuciaMagnaldo, ThierryAlvarez, MartinaMagnusson, Karl-EricAlvarez-Dominguez, CarmenMagri, GiuliaAlvisi, Maria FrancescaMahadevan, DarukaAlzahrani, NayefMahajna, JamalAmantini, ConsueloMahesh, GuruswamyAmbrosini, GraziaMai, SabineAmmazzalorso, AlessandraMai, SabineAmmendola, RosarioMaia, CláudioAmodio, NicolaMaier, PatrickAmpollini, LucaMaina, FlavioAmrutkar, ManojMaiorano, DomenicoAn, Jeung HeeMaiti, AparnaAnamthathmakula, PrashanthMaity, SankarAnand, KartikMajd, NazaninAnania, Maria ChiaraMajello, BarbaraAndera, LadislavMajhen, DragomiraAnderson, PeterMajhi, PrabinAndl, Claudia D.Majka, MarcinAndo, KoichiMajkowska-Pilip, AgnieszkaAndreuzzi, EvaMajumder, MousumiAndrikopoulos, PetrosMajumder, MrinmoyeeANDRYSIK, ZdenekMakena, Monish RamAnel, AlbertoMalapelle, UmbertoAngelousi, AnnaMalapelle, UmbertoAngelucci, AdrianoMalarkannan, SubramaniamAngileri, FilippoMale, Victoria H.Angrand, Pierre-OlivierMalekigorji, MaryamAnguille, SébastienMalik, RavinderAniello, FrancescoMalleo, GiuseppeAnkathil, RavindranMalouf, Gabriel G.Anker, StefanMalpeli, GiorgioAnsa, BenjaminMammadova-Bach, ElminaAnsorena, EduardoMamotte, Cyril D.Antognelli, CinziaManca, AntonellaAntón, Inés M.Mancuso, AndreaAntonarakis, EmmanuelManders, PeggyAntonios, GiakountisManes, SantosAntoniou, MichaelMangaonkar, AbhishekAnwer, FaizMangia, AnitaAo, GeyouMangla, AnkitAoki, MasahikoMango, LucioAoki, MasahiroMangogna, AlessandroAparicio Blanco, JuanMani, SridharApostolopoulos, VassoMann, MatiAppetecchia, MarialuisaManne, UpenderApplebaum, MarkMannelli, GiudittaAqil, FarrukhMannelli, MassimoAragonés, JuliánManning, Amity L.Aran, GemmaManov, IrenaArandjelović, OgnjenMantamadiotis, TheoArcangeli, AnnarosaMantamadiotis, TheoArcucci, AlessandroManzotti, GloriaArczewska, KatarzynaMarampon, FrancescoAref, Amir RezaMarampon, FrancescoArena, SabrinaMarcel, VirginieArend, Rebecca C.Marchant, Jonathan S.Ariga, KatsuhikoMarchbank, KevinArlt, AlexanderMarchetti, AlessandraArmellini, EliaMarchini, CristinaAronson, Daniel C.Marcovici, GenoArshad, NajlaMarengo, BarbaraAsgharian, HosseinaliMarí Alexandre, JosepAshaie, MaeirahMariadason, JohnAshihara, EishiMarian, BrigitteAshrafian Bonab, MaziarMariani-Costantini, RenatoAsim, MohammadMarín De Evsikova, CaralinaAsnaghi, LauraMarin, SilviaAsquith, Christopher R. M.Marín-Ramos, Nagore I.Assenat, EricMariotto, ElenaAstbury, StuartMarjanović, MarkoÅström, PirjoMarkan, Kathleen R.Attanasio, ChiaraMarkiewicz, AleksandraAttri, PankajMarkopoulos, AnastasiosAudet-Walsh, ÉtienneMarkopoulos, GeorgiosAuletta, LuigiMarkus, KrebsAushev, VasilyMármol, InesAvalle, LidiaMarotta, ClaudiaAvila, MatiasMarracci, SilviaAvtanski, DimiterMartell, KevinAwada, GilMartello, GrazianoAwasthi, NiranjanMartellucci, StefanoAydin, YucelMartens, John WMAye, JamieMartial, SoniaAylon, YaelMartignoni, GuidoAyuso, José MaríaMartin, JessicaAyyar, Balasubramanium VijayalakshmiMartin, PatriciaAzab, Abdel KareemMartín-Antonio, BeatrizAzarnia Tehran, DomenicoMartín-Antonio, BeatrizAzer, Samy A.Martinelli, PaolaAzhdarinia, AliMartínez, AlbertoAzimi, ImanMartinez, MagalyAzizi, EbrahimMartínez-Carmona, MarinaAzizkhan-Clifford, JaneMartínez-Soler, FinaAzoitei, NinelMartinez-Useros, JavierAzuma, MizukiMartinez-Zubiaurre, ÍñigoAzuma, ToshifumiMartin-Fontecha, AlfonsoAzzariti, AmaliaMartinho, OlgaBaba, HideoMartini, AlbertoBaba, TsukasaMartini, AndreaBaba, YuhMartins, AlbinoBach, Duc-HiepMartins, Andre F.Backman, SamuelMartins, Sandra Fátima FernandesBadgwel, BrianlMartinson, HollyBado, IgorMartín-Vasallo, PabloBadur, MehmetMartner, AnnaBae, Yong-SooMarty, CarolineBagci, UlasMarverti, GaetanoBaglieri, JacopoMarverti, GaetanoBagnard, DominiqueMary, DidierBagnoli, MarinaMas, EricBaguley, BrentonMasago, KatsuhiroBai, Li-YuanMasalova, Olga V.Bailly, ChristianMascitti, MarcoBailly, ClementMascitti, MarcoBaiocchi, MartaMascitti, MarcoBajaj, JeevishaMasel, Eva KatharinaBajusz, DávidMasjkur, Jimmy RusdianBajwa, AmandeepMasłyk, MaciejBakator, MihaljMasood, AshiqBaker, MatthewMasquida, BenoitBakht, MartinMastronuzzi, AngelaBalaj, LeonoraMasuda, MuneyukiBalalaeva, Irina V.Masui, KentaBalan, SreeKumarMatacchione, GiuliaBalasubramanian, SabaMatarrese, PaolaBalázs, MargitMatei, DanielaBalcerczak, EwaMaterin, Miguel A.Balcerczyk, AnetaMatesic, Lydia E.Baldassarre, GustavoMatheu, AnderBaldi, AlfonsoMatikas, AlexiosBaldini, NicolaMatin, AbdulBallarini, FrancescaMato Prado, MireiaBallester, Pedro J.Matos, PauloBalmus, GabrielMatozaki, TakashiBamdad, CynthiaMatsumori, TomoakiBanciu, ManuelaMatsumoto, KunioBancos, IrinaMatsuo, KojiBanerjee, AditiMatsushita, KazuyukiBanerjee, DebabrataMatsuzaki, ShinyaBanerjee, DipakMattei, VincenzoBanerjee, Hirendra NathMatteucci, ClaudiaBanerjee, KasturiMatthews, QianaBanerjee, SouravMattoscio, DomenicoBanerji, VershaMatulić, MajaBaniahmad, AriaMaugeri, Andrea GiuseppeBaniahmad, AriaMaugeri, MarcoBanito, AnaMaurel, JoanBánki, ZoltánMaurya, ShailendraBanks, LoriMavila, NirmalaBann, DarrinMavria, GeorgiaBao, YongpingMavrogonatou, EleniBaracos, VickieMayan, Maria D.Baranova, AnchaMazat, Jean-PierreBaras, Alexander S.Mazur-Bialy, Agnieszka IrenaBarazzuol, LaraMazza, TommasoBarbagallo, DavideMazzanti, MicheleBarberis, MassimoMazzoccoli, GianluigiBarbet, JacquesMazzoni, MaraBarbieri, AntonioMcBrayer, SamuelBarczak, WojciechMcClelland, SarahBardella, ChiaraMcCoy, MelanieBaregundi Subbarao, RaghavendraMcDonnell, AlisonBaritaki, StavroulaMcGonnell, Imelda M.Barlow, JacquelineMcGovern, JacquiBarnet, Megan B.McGowan, EileenBarone, InesMcGregor, Stephanie M.Barr, Martin P.McHenry, PeterBarrea, LuigiMcKenna, SharonBarreiro, EstherMcLeer-Florin, AnneBarrero, Maria J.McLeod, GeriBarresi, ValeriaMcSorley, Stephen T.Barrott, JaredMears, JasonBartee, EricMechtcheriakova, DianaBarth, BrianMedas, FabioBartneck, MatthiasMeder, LydiaBasak, DebasishMederacke, IngmarBasilico, ClaudioMedová, MichaelaBasilico, CristinaMedová, MichaelaBasini, GiuseppinaMeer, MargaritaBaskin, David S.Meerang, MayuraBasrai, MuniraMeerson, AriBastiaansen, Jessica A.M.Meeusen, ElsBasu, AlakanandaMehariya, SanjeetBasu, ArnabMehedi, MasfiqueBatish, MonaMehra, RaneeBatista, Miguel Luiz JrMehrabi, ArianebBatsios, PetrosMehta, Akul Y.Batta, KiranMehta, MeghnaBattaglia, AlessandraMehta, ParmenderBattistelli, CeciliaMehta, SunaliBauer, JohannMeier, Kathryn E.Bauleo, LisaMeierhofer, DavidBaxter, RobertMeldolesi, JacopoBazhin, Alexandr V.Meleady, PaulaBazzichetto, ChiaraMelenhorst, Jan JosephBeasley, ShannonMelillo, Rosa MarinaBeberok, ArturMelo, Sonia A.Bechmann, NicoleMemon, TosifaBecker, ThereseMendelsohn, Robin B.Beckner, Marie E.Mendogni, PaoloBednarska-Szczepaniak, KatarzynaMenendez, Sofia T.Bednarski, Patrick J.Menes, Tehillah S.Beenakker, Jan-WillemMénétrier-Caux, ChristineBehling, FelixMeng, XiangbingBehrens, JürgenMercado-Shekhar, KarlaBehrmann, IrisMercatali, LauraBeier, UlfMercer, CarolBeisswenger, ChristophMerchant, MichaelBekiaris, VasileiosMeriggi, PaoloBelardelli, FilippoMerino Peláez, GraciaBellstedt, PeterMerino, SoniaBel’skaya, LyudmilaMeroni, GermanaBeltran, ChrisMesker, Wilma E.Benada, JanMessaritakis, IppokratisBen-Eliyahu, ShamgarMesserli, Shanta M.Ben-Eliyahu, ShamgarMessersmith, AmyBenesch, MatthewMessiha, HadiBenevolenskaya, ElizavetaMetzeler, KlausBenistant, ChristineMeyer, Hans-JonasBenjamin, DonMeyerholz, David K.Benna, ClaraMezencev, RomanBennett, Dorothy C.Mian, HiraBennett, PeterMiazek, ArkadiuszBenoist, HervéMichaeu, OlivierBenovic, Jeffrey L.Michallet, Marie-CécileBensadoun, René-JeanMichalopoulos, Nikolaos V.Ben-Sahra, IssamMicheau, OlivierBentel, Jacqueline M.Michelhaugh, Sharon KayBento, CelesteMichelle, PalmieriBenzakour, OmarMichiels, CarineBeretta, LauraMierke, ClaudiaBernardes, NunoMigliaccio, AntimoBernardini, GiuliaMikami, ShujiBernardo, CarinaMikami, ShujiBernatík, OndřejMikelis, ConstantinosBerndt, Michael C.Mikropoulos, ChristosBertagnolo, ValeriaMikula, MarioBertero, LucaMilavetz, BarryBertherat, JérômeMilczarek, MałgorzataBertoli, GloriaMilella, MicheleBertoni, FrancescoMiles, Wayne OwenBertoni, FrancescoMiletic, HrvojeBertrand, CzarnyMiller, Anthony B.Bessette, BarbaraMiller, JeffreyBest, Oliver GilesMiller, KyleBeswick, Ellen J.Miller, PhilipBetancourt, Taniamiller, vandanaBetts, Brian ChristopherMiller, VandanaBeutner, DirkMills, KenBeyaert, RudiMiloso, MariarosariaBeyer, Eric C.Mimura, JunseiBeyer, KatharinaMinami, YasunoriBezombes, ChristineMinichsdorfer, ChristophBhagirath, DivyaMinicozzi, PamelaBhat, KrishnaMinniti, GiuseppeBhattacharjee, SonaliMino-Kenudson, MariBhosale, PriyaMinucci, AngeloBi, ChengfengMinucci, SaverioBi, XinMiotto, BenoitBianchi, FabrizioMir, HinaBianchini, FrancescaMiranda, EnriqueBianco, RobertoMiranda, Joana P.Bidère, NicolasMiranda-Gonçalves, VeraBidet, YannickMirshahi, MassoudBidoli, EttoreMisawa, KiyoshiBiersack, BernhardMiserocchi, GiacomoBijlsma, Maarten F.Mishra, AvanishBilbao, DanielMisso, GabriellaBilek, RadovanMitra, SumeghaBillotey, ClaireMitsuhashi, AkiraBindels, LaureMiwa, ShinjiBinder, HansMiyagaki, TomomitsuBins, Adriaan D.Miyagawa, KiyoshiBiondi, AntonioMiyamoto, David T.Birembaut, PhilippeMiyata, YoshihikoBishehsari, FarazMiyazaki, MasaruBishop, KarenMiyazawa, KeijiBisio, AlessandraMiyazawa, MasaakiBiswas, SantanuMiyoshi, HiroakiBiswas, TithiMiyoshi, NorioBizzarri, MarianoMo, FeiBjarnason, GeorgMo, YanyuanBlache, PhilippeMobashsher, Ahmed ToahaBlack, JenniferMocarski, EdwardBlackburn, JessicaMoccia, FrancescoBlakely, Andrew M.Modak, ShakeelBlandino, GiovanniModenese, AlbertoBlonska, MarzennaModjtahedi, HelmoutBoard, PhilipModos, DezsöBocharov, GennadyMody, Christopher H.Bochicchio, FrancescoMody, HardikBock, Karl WalterMoens, Ugo LionelBöckmann, LarsMoertel, ChristopherBodenstine, ThomasMoffitt, RichardBodis, StephanMohamedali, KhalidBödör, CsabaMohammad, Ramzi M.Boehm, DanielaMohammed, AltafBoeri, MattiaMohammed, AltafBoerma, MarjanMohanty, VakulBoichuk, SergeiMohile, Supriya G.Boiko, Alexander D.Mohr, AndreaBoisgerault, NicolasMok Yang, YoungBojarczuk, KamilMok, SamuelBölke, EdwinMoldoveanu, TudorBommareddy, PraveenMolena, DanielaBonanno, ElenaMolenaar, Remco J.Boncela, JoannaMolina-Cerrillo, JavierBoncoeur, EmilieMolinari, ChiaraBondoc, AlexanderMolina-Vila, Miguel ÁngelBonetto, AndreaMøller, PålBonne, NicolasMonaco, MarieBonnelye, EdithMoncayo, RoyBonnet, Pierre-AntoineMoncharmont, BrunoBonomi, PhilipMong, Mei-ChinBooka, EisukeMonga, VarunBoon, ChengMongiat, MaurizioBordonaro, MichaelMongin, AlexanderBork, KayaMontagnani Marelli, MarinaBornhorst, MiriamMontazeri Aliabadi, HamidrezaBorodkina, AleksandraMonteiro, AlvaroBoros, LászlóMonteiro, Ana CarolinaBorrego, FranciscoMonteith, GregBose, TanimaMontemurro, FilippoBossenmeier, BirgitMontenegro, LuciaBosserhoff, AnjaMontesarchio, DanielaBostjancic, EmanuelaMontfort, AnneBotti, GerardoMontgomery, McKaleBotton, ThomasMontopoli, MonicaBoubchir, LarbiMorales, JulioBoublikova, LudmilaMorandi, LucaBoulaiz, HouriaMorbidelli, LuciaBoumber, YanisMorbidelli, LuciaBourneuf, EmmanuelleMoreaux, JeromeBoussios, StergiosMoreira, DaysonBoutros, JacquesMoreira, Leticia Z.Bowen, Thomas ScottMorel, SandrineBowles, Kristian M.Moreno, Carlos S.Bozec, AlexandreMoreno, J.J.Bozec, AlineMoreno-Loshuertos, RaquelBrabletz, ThomasMoretti, MartaBracken, CameronMorgan, EthanBraconi, ChiaraMorgan, Iain M.Brady, GarethMorgan, MichaelBragazzi, Nicola LuigiMorgan, RichardBrancolini, ClaudioMori, YasuoBrandell, Richard RosenquistMoritz, Michael L.Brandi, GiovanniMorland, BruceBrands, Roman C.Moro, LoredanaBraun, ThorstenMoro, LoredanaBraunstein, Evan M.Moroishi, ToshiroBravatà, ValentinaMorreau, HansBravo, JeronimoMorris, AndrewBrechbuhl, HeatherMorris, DonBreitenfeld Granadeiro, LuizaMorris, Don G.Brekken, Rolf A.Mortimer, PeterBrenner, Annette K.Morton, Lindsay M.Brenner, Chad J.Mosca, EttoreBrenner, J. ChadMosialos, GeorgeBresson-Bepoldin, LaurenceMoskowitz, Alison J.Bret, CarolineMoss, PaulBrett, ThomasMotegi, AtsushiBrigante, GiuliaMou, HaiweiBrignole, ChiaraMoulin, AlexandreBrillanti, StefanoMountzios, GiannisBrindley, DavidMourah, SamiaBrindley, DavidMouw, Kent W.Brink, PeterMuallem, Mustafa ZelalBrissot, ÉoliaMucignat, CarlaBristol, Molly LMudryj, MariaBriz, OscarMueller, MathiasBrizuela, LeyreMuhammad, Jibran SualehBrodosi, LuciaMuir, AlexanderBroggi, GiuseppeMujtaba, ShirazBrohl, Andrew S.Mukaida, NaofumiBronte, EnricoMukaida, NaofumiBronte, FabrizioMukherjee, AbirBrooke, GregMukherjee, RajibBrooks, JenniferMukherjee, SarbajitBroos, KatrijnMukherjee, SomnathBros, MatthiasMukherjee, SudipBrosens, Lodewijk A.A.Mukherjee, SudipBrouwers, Jos F.Mukherjee, SudipBrown, JamesMukhopadhyay, SumanBrown, Kai M.Mulcahy Levy, Jean M.Brown, KristinMulder, FritsBrownell, IsaacMulherkar, Shalaka AjitBrozek-Pluska, BeataMuller, Patricia A. J.Brozyna, AnnaMüller-Klieser, WolfgangBrummer, TilmanMüller-Newen, GerhardBrunet De La Grange, PhilippeMullin, StephenBrunetti, OronzoMultani, AshaBrunetti-Pierri, NicolaMunaron, LucaBruserud, ØysteinMuniraj, NethajiBruserud, ØysteinMuniyan, SakthivelBruzzone, SantinaMuñiz, PilarBuchanan, PaulMunkley, JenniferBuchbinder, ElizabethMuñoz Caffarel, MaríaBuckanovich, RonaldMuñoz, PurificaciónBuckanovich, RonaldMunshi, AnupamaBuda, GabrieleMurakami, YujiBuddaseth, SalmaMuralidharan, PriyaBudillon, AlfredoMurata, TakayukiBudzevich, Mikalai M.Murata, TakayukiBui, Jack D.Murdaca, GiuseppeBujanda, LuisMurph, Mandi M.Bujanda, LuisMurphy, AndrewBujko, MateuszMurphy, Daniel J.Bulboacă, Adriana ElenaMurray, DavidBulk, EtmarMurray, PaulBuonaguro, LuigiMusani, VesnaBurbury, Kate L.Muscarella, Lucia AnnaBurchell, JoyMusti, Anna MariaBurdak-Rothkamm, SusanneMustjoki, SatuBurdette, Joanna E.Musumeci, GiuseppeBurger, Michael CMus-Veteau, IsabelleBurian, EgonMuthupillai, RajaBurke, DermotMutsaers, Steven E.Burnette, Pearlie K.Mutti, LucianoBurnier, JuliaMuzii, Vitaliano FrancescoBurnouf, ThierryMuzio, Lorenzo LoBurstyn-cohen, TalMyllymaa, SamiBuscail, LouisMymryk, JoeBüsselberg, DietrichMyneni, AjayBusson, PierreMythreye, KarthikeyanButi, LudovicoMyung, Seung-jaeButi, LudovicoNachmias, BoazButt, Julia A.Nadel, HelenBuzzá, Hilde HarbNadiminty, NagalakshmiByrne, HughNagai, HidenariByun, SanguineNagai, KatshitoC. Hirbe, AngelaNagano, TatsuyaCabrini, GiulioNagarajah, JamesCacalano, NicholasNagaraju, Ganji PurnachandraCacchione, StefanoNagelkerke, AnikaCaceres, SaraNaghibalhossaini, FakhraddinCadamuro, MassimilianoNagineni, ChandraCaers, JoNagpal, SeemaCai, DeminNaiki, TakuCai, LishengNair, Gopika G.Caiazzo, ElisabettaNaito, TateakiCaisová, VeronikaNaito, YoichiCaizzi, LiviaNajafova, ZeynabCakstina, IneseNajar, AmerCalabrese, GiovannaNakagawa, TakashiCalabrò, ViolaNakagawa, TakuroCalapre, LeslieNakai, AkiraCaldwell, Julie M.Nakai, YousukeCaliò, AnnaNakajima, MasanobuCalistri, DanieleNakajima, TakahiroCalogero, AntonellaNakamura, KyoheiCalorini, LidoNakamura, MasatoCaltabiano, RosarioNakamura, TomokiCalvisi, Diego F.Nakanishi, YokoCalvisi, Diego FrancescoNakano, KeisukeCameron, AngusNakano, KenjiCammas-Marion, SandrineNakano, ToshiakiCampagna, SaraNakao, KohshiroCampana, DavideNakashima, SouichiCampana, Luca GiovanniNakashima, YoshikiCampos, EricNakata, WataruCamps, JordiNakayama, HironaoCanal, CristinaNakayama, KohCandéias, SergeNam, Jeong-SeokCandido Carvalho, KatiaNam, Jin-WuCanevari, SilvanaNamikawa, KenjiroCanfield, ScottNan, HongmeiCanter, Robert J.Nanba, KazutakaCantile, MonicaNandana, SrinivasCantley, Melissa D.Nandi, SaikatCantor, Jason R.Naponelli, ValeriaCapasso, RaffaeleNappi, LuciaCapece, DariaNariai, TadashiCapilla-Capilla, VivianNarod, Steven A.Capitini, Christian M.Nasarre, PatrickCaputo, Sandrine M.Naseer, Prof. NomanCarafa, VincenzoNass, NorbertCarbone, MicheleNassar, Zeyad D.Caretta, AntonioNassiri, MehdiCaria, PaolaNastuta, Andrei VasileCarlisi, DanielaNastuta, Andrei VasileCarlo, MariaNatarajan, Sathish KumarCarnero, AmancioNateri, Abdolrahman ShamsCarnero, AmancioNath, NiharikaCarnesecchi, JulieNath, ShubhankarCaron, Joan McIntyreNath, ShubhankarCarpenter, RichardNatkunam, YasodhaCarrano, AnnaNatrajan, Rachael C.Carrara, MariaNatsumeda, ManabuCarrer, AlessandroNaumann, PatrickCarreras, JoaquimNavarra, MicheleCarrier, AliceNaves, ThomasCarrier, PaulNavran, ArashCarro, MariaNawaz, MuhammadCarruthers, Ross D.Nawaz, MuhammadCarter, WayneNawrocki, Steffan T.Carullo, GabrieleNawroth, RomanCaruntu, ConstantinNayak, TapanCarvajal, RichardNaylor, KariCarvalho, BeatrizNchinda, Godwin W.Carvalho, Robson FranciscoNduom, EdjahCasadei-Gardini, AndreaNedeljković, MilicaCasanova, EmilioNees, MatthiasCasanova, LlanosNegri, RodolfoCasanova, MichelaNehme, AliCasanovas, OriolNelson, Peter J.Cascella, MarcoNephew, Kenneth PCascione, LucianoNero, CamillaCaserta, DonatellaNettersheim, DanielCash, HannesNeubauer, HeidiCaspari, ThomasNeubig, Richard R.Cassinelli, GiulianaNeuhaus, JochenCassoni, PaolaNeureiter, DanielCassoux, NathalieNevalainen, MarjaCastelli, VanessaNeve, BernadetteCastellón, Enrique A.Newton, JasonCastoria, GabriellaNewton-Bishop, JuliaCastresana, JavierNg, AndyCastriconi, RobertaNgamcherdtrakul, WorapolCastro-Giner, FrancescNguyen, Andrew V.Castro-Vega, Luis JaimeNi, DalongCasuso-Pérez, RafaelNi, YichengCatacuzzeno, LuigiNichols, Romaine C.Catalano, OnofrioNicolaides, TheodoreCatanzaro, GiuseppinaNicoletti, FerdinandoCaulfield, Thomas R.Nicolini, AndreaCaux, ChristopheNicoll, Amanda J.Cava, ClaudiaNicolle, RemyCavalloni, GiulianaNieberler, MarkusCavanaugh, JaneNielsen, Finn CiliusCazzador, DiegoNiemira, MagdalenaCeci, FrancescoNien, Hsin-HuaCeckova, MartinaNierkens, StefanCeletti, AngelaNierkens, StefanCelia-Terrassa, ToniNifli, Artemissia-PhoebeCelsi, FulvioNigris, Filomena DeCen, OsmanNijhof, IngerCeppi, PaoloNijland, MarcelCerchia, LauraNikfarjam, MehrdadCereda, MatteoNiklison Chirou, Maria VictoriaCeresa, BrianNiles, Richard MCeribelli, MicheleNilsson, Inga-LenaCerrada, Maria LuisaNilsson, JonasCerrito, Maria GraziaNilubol, NarisChaganty, BharatNilvebrant, JohanChakrabarti, KausikNirala, NirajChakraborty, SayanNishida, ShozoChakravarty, ShatadruNishikawa, DaisukeChalela, RobertoNishikawa, JunChalla, DilipNishikawa, TetsuoChallagundla, KishoreNitulescu, MihaiChalmers, AnthonyNjar, Vincent C.O.Chalmers, Jeffrey J.Nobis, MaxChamaraux-Tran, Thiên-NgaNoel, GeorgesChan, AdenNoghero, AlessioChan, AlexanderNoguchi, MasayukiChan, AndrewNolte, IngoChan, AnitaNonaka, TaichiroChan, David WaiNonami, AtsushiChan, Kuan RongNonnekens, JulieChan, MichaelNoonepalle, Satish KumarChandra, DhyanNoorani, ImranChandrasiri, UpekshaNor Eddine, SounniChang, Chia-cheNoren Hooten, NicoleChang, Chien-ChungNoríaki, TomuraChang, HongNorihiko, NaritaChang, Hsiu-HaoNormanno, NicolaChang, Hsueh-WeiNorton, Kerri-AnnChang, HyunNottegar, AlessiaChang, John C.Noubissi, Felicite KamdemChang, Li-ChingNovakovic, BorisChang, Ling-ChuNovickij, VitalijChang, Tien-ChunNowak-Sliwinska, PatrycjaChang, Wen-WeiNowinska, KatarzynaChang, Yih-LeongNowis, DominikaChang, Yu-JiaNowotarski, ShannonChao, Wei-TingNozaki, MasamiChapoval, Svetlana P.Nüesch, JürgCharles Jacob, Harrys KishoreNúñez Torres, María IsabelCharles, KellieNúñez-Iglesias, María JesúsCharlton, ClivelNunziata, AngelinaCharmsaz, SaraNuovo, Gerard JamesChastre, ÉricNylander, KarinChatterjee, IshitaNylander, KarinChatterjee, JhinukO’Hanlon, ShaneChatterjee, NimratO’Sullivan, Joe M.Chatzistamatiou, KimonOakhill, JonChauhan, MunishObara-Michlewska, MartaChauhan, SubhashObermayr, EvaChaurand, PierreObinata, DaisukeChauvie, StephaneOcchi, GianlucaChen, ArgonOchiya, TakahiroChen, ChanghanOchs-Balcom, HeatherChen, Chien-ChinOcio, Enrique MChen, Chi-YuanOćwieja, MagdalenaChen, Chun-JungOehme, InaChen, Chun-LiangOffodile, AnaezeChen, DechanOgasawara, ToruChen, Georgia ZhuoOgino, TakashiChen, GuoxunOgris, ManfredChen, HongwuOh, Eok-SooChen, HuabiaoO’Hagan, HeatherChen, HuanhuanOhara, ToshiakiChen, Kuan-FuOhashi, RiukoChen, LiangOhe, KenjiChen, LongwenOhguchi, HirotoChen, Mei-ChuanOh-Hohenhorst, Su JungChen, Mei-RuOhkuri, TakayukiChen, Pai-ShengOhmoto, AkihiroChen, PeiwenOhtsuka, MasayukiChen, QiOing, ChristophChen, ShukunOkada, MasahiroChen, SonghaiOkada, MasashiChen, SuzieOkada, TomoyoChen, TaoOkamoto, TakayukiChen, Wei R.Okamura, RyosukeChen, Wei-YuOkegawa, TakatsuguChen, XiangOkui, TatsuoChen, XinOkuma, YusukeChen, XuOliva, StefaniaChen, Yih-FungOliveira, PatríciaChen, YuOliveira, Paula A.Chen, Yu-JenOliveira-Ferrer, LeticiaChen, Yun-JuOlofsson Bagge, RogerChen, ZhenOlson, Thomas L.Chen, ZhengOnali, PierluigiChen, ZhihangO’Neill, AllisonChen, ZhitongO’Neill, EricCheng, HailiOng, QunxiangCheng, HeatherOnken, MichaelCheng, Heung-ChinOno, TakashiCheng, JiongjiaOO, Joo HyunCheng, KeOoi, AiksengCheng, Kuang-HungOosterwijk, EgbertCheng, Ming-HueiOrimo, AkiraCheng, Shih-PingOrlandi, EsterCheng, Tsu-YaoOrouji, EliasCheng, XinlaiOrth, James D.Cheng, Yuen YeeOrzechowski, ArkadiuszCheon, Dong-JooOrzechowski, ArkadiuszCheon, Yong-PilOsaki, TomohiroCheung, AdaOsaki, TomohiroCheung, Siu TimOse, JenniferChew, ValerieO’Shaughnessy, RyanChhabra, GaganOshima, TadayukiChhabra, GaganOstroumova, EugeniaChhabra, YashOta, YosukeChiam, KarenOtero, CarolinaChiang, Hsiu-MeiOtowa, YasunoriChiang, Ying-ChengOtsuki, TakemiChiantore, Maria VincenzaOttaiano, AlessandroChiappinelli, KatherineOtto, AngelaChicas-Sett, RodolfoOvchinnikov, Lev P.Chien, Chun-RuOve, RogerChien, JeremyOw, ThomasChina, ArnabOzaki, IwataChinegwundoh, FrancisOzaki, ToshinoriChinen, Ludmilla Thomé DomingosOzsvari, BelaChintala, SreenivasuluPabst, ThomasChiodoni, ClaudiaPachmann, KatharinaChiovato, LucaPacifico, Francesco M.Chirackal, SintosebastianPackiam-Dayalan, AntonyChirumbolo, SalvatorePaderno, AlbertoChiu, Ing-MingPagano, AlessandraChiu, Yi-HanPage, AndrewChlichlia, KaterinaPages, MelanieCho, Hea-YoungPaggetti, JérômeCho, HyosunPagni, FabioCho, Je-YoelPai, KedarCho, Kang SuPaic, FraneCho, MayPainschab, MatthewCho, Ssang-GooPal Choudhuri, ShreoshiCho, Yong BeomPal, DebjaniCho, YongyeonPalacios, FlorenciaChobot, VladimírPaleari, LauraChoi, Doo HoPalena, ClaudiaChoi, Eun HaPalese, Luigi LeonardoChoi, JunjeongPalko-Labuz, AnnaChoi, Seok KiPallavi, ManralChou, C. JamesPalle, KomaraiahChou, Chih-HungPaller, Channing JudithChou, Wen-ChiPallottini, ValentinaChou, Wen-ChienPalmer, ChristopherChouaib, SalemPalmieri, CamilloChoudhary, SanjeevPalmieri, DarioChoulier, LaurencePalmqvist, LarsChovanec, MichalPaluszczak, JarosławChowdhury, Ezharul HoquePan, ChenyiChristensen, Jørn BPan, Mei-RenChu, Ching-LiangPan, MingguiChu, Pei-YiPan, ZuiChu, XianpingPanabieres, CatherineChuang, Show-MeiPanchapakesan, BalajiChuang, Wen-YuPancione, MassimoChueh, Anderly C.Pandey, SudipChuffa, Luiz GustavoPanikkanvalappil, Sajanlal R.Chulanov, VladmirPannequin, JulieChuma, MakotoPanse, Gauri G.Chung, Byung MinPantham, PriyadarshiniChung, ChaeukPantziarka, PanChung, Chin HaPaoli, PaoloChung, Ching-HuPaolillo, MayraChung, Eun JooPaolini, FrancescaChung, JinsooPaone, AlessioChung, JunhoPapa, AlfredoChurchman, Michelle L.Papa, AntonellaChuu, Chih-PinPapaccio, FedericaChwistek, MarcinPapageorgis, PanagiotisCiaglia, ElenaPapavassiliou, Athanasios GCiarrocchi, AlessiaPapi, AlessioCiccarelli, RenataPapp, BélaCiccolini, JosephPappas, DimitriCicenas, JonasPaquette, BenoitCichorek, MiroslawaParadise, Brooke D.Cifuentes-Rius, AnnaParanjape, AnuragCimmino, AmeliaParashar, DeepakCimmino, FloraParat, Marie-OdileCippitelli, MarcoParayath, NehaCiregia, FedericaPardini, BarbaraCirone, MaraParekh, AnantCisowski, JaroslawParet, ClaudiaCiszewski, WojciechParis, JuanCiszewski, Wojciech MichałPark, Chan HeunCitro, SimonaPark, Eun-youngCittaro, DavidePark, Hae RyounCitterio, DavidePark, Hwan-WooClausen, JohannesPark, JohnClawson, Gary A.Park, Jong HoonClifford, GaryPark, Jong KookCliment, MontserratPark, Jong KookCochrane, Dawn RPark, Jong KookCoelho-Silva, JuanPark, KeunchilCohen, RomainPark, Shin-HyungCoin, FrédéricPark, Sung-GyooColeman, Helen GParolini, CinziaCollavin, LicioParra Cuentas, Edwin RogerCollery, PhilippeParrington, JohnCollura, AdaParsons, JasonColoff, JonathanParsons, Lauren N.Coluccia, MauroParthasarathy, SrinivasColumbano, AmedeoPasanisi, PatriziaCombes, Jean DamienPasquet, Jean-MaxCommisso, CosimoPatankar, ManishCondello, MariaPatel, Akash J.Condello, SalvatorePatel, Girijesh KumarCondorelli, GerolamaPathak, YashwantConlan, StevenPathania, RajneeshConn, SimonPatil, RameshwarContaldo, MariaPatrícia, BarrosCook, KristinaPatrignani, PaolaCooke, MarianaPatruno, MarcoCoppedè, FabioPatwardhan, Parag P.Cora’, DavidePausch, Thomas.Corbo, ClaudiaPause, ArnimCordani, MarcoPavankumar, TheethaCordenonsi, MichelangeloPavlidis, EfstathiosCordero, OscarPavlik, EdwardCordo Russo, Rosalia I.Pavlova, NatalyaCormio, AntonellaPawar, SnehalataCornillet, MartinPayne, AnnetteCorre, IsabellePazgan-Simon, MonikaCorrea, Maria ElviraPécheur, Eve-IsabelleCorrenti, MargheritaPecqueur, ClaireCorsello, Salvatore MariaPeddle-McIntyre, Carolyn J.Corsi, FabioPedroni, MonicaCortes-Dericks, LourdesPeinemann, FrankCortese, KatiaPeitzsch, ClaudiaCorti, AlessandroPejoski, DavidCosenza, MariaPellicano, RinaldoCosta Gomes, BrunoPemov, AlexanderCosta-Almeida, RaquelPeña, CristinaCostantini, LaraPena, Maria MarjoretteCosta-Silva, BrunoPenfornis, PatriceCostelli, PaolaPeng, AiminCostoya, Jose A.Peng, HanCote, Gregory M.Peng, Xianlu LauraCotelle, PhilippePenna, FabioCotta, ClaudiuPennacchioli, ElisabettaCoupland, Lucy A.Pennock, NathanCoupland, SarahPentimalli, FrancescaCoussons, PeterPenuela, SilviaCowden Dahl, KarenPeperzak, VictorCox, Charles D.Peppelenbosch, M.P.Crabb, JohnPeraldo-Neia, CaterinaCrawford, LindseyPeraldo-Neia, CaterinaCrawford, LisaPerbellini, LuigiCremonesi, FaustoPercesepe, AntonioCrespan, EmmanuelePerche, FédéricoCrinò, LucioPerea, JoséCristescu, RazvanPerego, MichelaCronauer, Marcus V.Perego, PaolaCroset, MartinePereira, AllanCross, MichaelPérez Sánchez, HoracioCross, NicholasPérez, ConcepciónCrucho, Carina Isabel CorreiaPérez-Campo, Flor MaríaCruickshank, Margaret EleanorPérez-Ortiz, José M.Crum, ChristopherPerez-Stable, CarlosCsordas, GyorgyPérez-Torras, SandraCuburu, NicolasPeri-Rotem, NitzanCui, TiantianPerkins, NeilCui, XiaojiangPero, RaffaelaCui, ZhibinPérol, DavidCulig, Zoran CuligPerri, FrancescoCurtale, GraziellaPerry, ChristopherCurtin, NicolaPersico, MarcelloCurtis, MarionPerucho, ManuelCurto, SergioPerumal, VanathiCutler, Mary LouPešić, MilicaCytlak, UrszulaPessina, AugustoCzarniecka, AgnieszkaPessina, FedericoCzyż, JarosławPessoa, Rodrigo SavioCzyż, JarosławPeter Sinn, HansCzyz, MalgorzataPethő, ZoltánD Prestwich, Robin JPetkov, Valentina I.D’Argenio, ValeriaPetralia, Maria CristinaD’Elios, Mario MilcoPetrenko, OleksiD’Orazi, GabriellaPetrillo, AngelicaDa Costa, Rui M. GilPetros, John A.Dabi, YohannPetrov, AntonDabrowiak, JamesPetrulionis, MariusDachineni, RakeshPeyruchaud, OlivierDafou, DimitraPezzuto, AldoDaftarian, PirouzPfeffer, UlrichD’Agostino, Vito GiuseppePfeffer, UlrichDahl, ChristinaPhan, KhaDahlhoff, MaikPhan, TienDahlmann, MathiasPhilipp, AlexanderDaidone, Maria GraziaPhillips, Kelly A.Daigaku, YasukazuPhillips, Mary E.Dakhlallah, DuaaPicard, DidierDal Col, JessicaPiccirillo, SaraDal Col, JessicaPiccolo, StefanoD’alessio, AlessioPichler, MartinDalla Palma, BenedettaPickering, Curtis R.Dalla Pozza, ElisaPiekiełko-Witkowska, AgnieszkaDalm, Simone U.Pierantoni, Giovanna MariaDamalanka, VishnuPierpaoli, ElisaDamaskos, ChristosPierzchalski, PiotrDamato, BertilPilarsky, ChristianD’Amico, Agata GraziaPiluso, GiulioDamm-Welk, ChristinePinheiro, MarinaDandawate, PrasadPinho, MarcoDandekar, GudrunPinter, MatthiasDangol, ManitaPinto, MafaldaDanielson, KirstyPinzani, PamelaDann, EldadPiotto, M. MartialD’Arcy, PadraigPiovesan, AlessandroD’Argenio, ValeriaPiplani, HonitDario Mandato, VincenzoPirtoli, LuigiDarling-Reed, Selina F.Pisapia, PasqualeDarwiche, KaidPisegna, Prof J.Das, GokulPitts, Todd M.Das, SudipPiulats, JosepDas, SunitPiva, RobertoDasgupta, ParamitaPiva, TerrenceDaskalakis, KosmasPivarcsi, AndorDass, JoshuaPiwowarczyk, KatarzynaDaubon, ThomasPlaitakis, AndreasDavalos-Salas, MercedesPlamper, MichaelaDavar, DiwakarPlassais, JocelynDave, SandeepPo, AgneseDavid, GregoryPocard, MarcDavid, MutchPoeta, Maria LuanaDavidson, MichaelPoettler, MarinaDavies, AndrewPoggi, AlessandroDavies, Kurtis D.Pohl, SebastianDavies, Michael J.Poirot, MarcDavis, Mellar P.Pojo, MartaDawson, Michelle R.Poku, Rosemary A.De Alarcon, Pedro A.Polakowski, Nicholas J.De Araujo, ElvinPoli, AlessandroDe Blank, Peter MatthewPoli, ValeriaDe Bock, Geertruida HendrikaPolimeni, AntonellaDe Bortoli, MichelePollock, RaphaelDe Bree, RemcoPollok, Karen E.De Brevern, Alexandre G.Polly, PatsieDe Carlo, FlaviaPoma, PaolaDe Caro, Maria Laura Del BassoPonnusamy, Thamarai (Suriyan)De Chiara, LorettaPonziani, Francesca RomanaDe Falco, ValentinaPonz-Sarvise, MarianoDe felice, FrancescaPoon, IvanDe Felice, MarioPopp, Henningde Franciscis, VittorioPópulo, HelenaDe Gruijl, FrankPorcellini, ElisaDe Jong, StevenPorgador, AngelDe Klein, AnneliesPorras, AlmudenaDe La Fouchardière, ArnaudPortella, GiuseppeDe La Serna, Ivana L.Posey, Avery D.De La Vega, LaureanoPosselt, GernotDe Lorenzo, Mariana SPotaczek, Daniel PDe Luca, AntonellaPotenza, NicolettaDe Martino, Maria CristinaPoulaki, VassilikiDe Matteis, Valeria Pouliopoulos, AntoniosDe Mattia, ElenaPouliot, FredericDe Melo-Diogo, DuartePouponnot, CelioDe Nola, RosalbaPourquier, PhilippeDe Pittà, CristianoPouvesle, Jean MichelDe Rasmo, DomenicoPouysségur, JacquesDe Re, VallìPowrózek, TomaszDe Rienzo, AssuntaPozzobon, MichelaDe Rosa, MarinaPrados, Michael D.De Rosa, VivianaPramanik, ArindamDe Sepulveda, PauloPrante, OlafDe Smaele, EnricoPratsinis, HarrisDe Summa, SimonaPrenen, HansDe Toni, EnricoPrescott, AlanDe Toni, LucaPresneau, NadegeDe Totero, DanielaPrévostel, CorinneDe Ville De Goyet, JeanPrieto, Victor G.De Vita, AlessandroPrimig, MichaelDe Voer, Richarda M.Principe, Maria Ilaria DelDe, PradipPrior, IanDeaglio, SilviaPrise, Kevin M.Dean, MichaelPrivat-Maldonado, AngelaDebeljak, NatašaProbert, ChrisDeben, ChristopheProchownik, Edward V.DECAENS, THOMASProcopio, GiuseppeDecaestecker, ChristineProescholdt, MartinDeCaprio, JamesProsperi, EnnioDecaudin, DidierProvot, SylvainDecaussin-Petrucci, MyriamPruszynski, MarekDechecchi, Maria CristinaPrzybyłek, MaciejDeel, MichaelPrzybyło, MałgorzataDeep, GaganPuca, LoredanaDeFilipp, ZachariahPuglisi, FabioDegraff, DavidPuhka, MaijaDegrassi, FrancescaPulford, KarenDehzangi, AbdollahPulido, RafaelDejaco, DanielPuliyappadamba, Vinesh ThidilDejardin, EmmanuelPulliero, AlessandraDel Ben, FabioPuls, FlorianDelgado, EsmeraldaPurohit, VineeD’Elios, Mario MilcoPurrello, MicheleDelisle, Jean-SébastienPusch, StefanDellaire, GrahamPusztaszeri, MarcDellis, OlivierPutluri, NagireddyDel-Rio Celestino, MercedesPuukila, StephanieDelyon, JuliePuxeddu, EfisioDeng, PengQi, HongDeng, RuixiaQian, ChunqiDeng, YoupingQian, WeiDeNicola, GinaQie, YaqingDenis, JeromeQin, Xian-YangDenis, SerenoQiu, XinDenoyer, DelphineQiu, YiDent, PaulQiu, ZhenDent, SusanQu, PengDepciuch, JoannaQuaini, FedericoDer Tuin, Karin VanQuante, MichaelDer Wekken, Anthonie VanQuaquarini, EricaD’Erme, MariaQuarello, PaolaDeRyckere, DeborahQuattrocchi, AnnalisaDeryusheva, SvetlanaQue, JennyDesai, DhimantQueiroga, FelisbinaDesantis, VanessaRabanal-Ruiz, YoanaDesbois-Mouthon, ChristèleRaber-Durlacher, Judith E.Descamps, GéraldineRacanelli, VitoDeshayes, FrédériqueRadchenko, Eugene V.Deshayes, SébastienRaderer, MarkusDeshpande, ShayuRadhakrishnan, PrakashDetours, VincentRadic, TanjaDeutz, NicolaasRadoul, MarinaDhakal, DipeshRaffaniello, Robert D.Dharmarajan, ArunRafiq, KhadijaDharmawardhane, SuranganieRager, OlivierDhawan, DeepikaRaghunandan, MayaDhungel, BijayRagni, EnricoDi Agostino, SilviaRagnini-Wilson, AntonellaDi Donato, MarziaRahib, LolaDi Fazio, PietroRahikkala, AnttiDi Francia, RaffaeleRahmathulla, GazanfarDi Franco, SimoneRahme, GilbertDi Giulio, Anna Maria Rai, PrakashDi Marcotullio, LuciaRai, SumitDi Martino, PieraRaimondi, LaviniaDi Napoli, AriannaRaimondo, StefaniaDi Nicola, MassimoRaingeaud, JoëlDi Paolo, AntonelloRaj, NitinDi Renzo, LiviaRajagopalan, AnugrahaDi Zazzo, ErikaRajas, OlgaDiana, PatriziaRajendra, ShanmugarajahDias-Ferreira, JoãoRajpoot, NasirDickerson, ErinRajpurohit, SurendraDickson, Paxton V.Rakic, JelenaDidiášová, MiroslavaRamaiahgari, Sreenivasa C.Didier, ChristineRamakrishnan Geethakumari, PraveenDiefenbacher, Markus E.Ramakrishnan, VijayDiehl, FrankRamalingam, NaveenDietrich, MartinRamani, KomalDietrich, OlafRamani, VijayDíez Valle, RicardoRamaraj, PanduranganDiez, OrlandRamasamy, MohankandhasamyDimanche-Boitrel, Marie-ThérèseRambow, FlorianDimos, KonstantinosRamchandani, DivyaDing, Wei-QunRampazzo, ElenaDiniz, Márcio AugustoRampias, TheodorosDinulescu, Daniela M.Rana, AjayDinulescu, Daniela M.Randall-Demllo, SarronDioguardi, Francesco S.Rangaswami, ArunDionisi, FrancescoRanieri, ElenaDiquigiovanni, ChiaraRanieri, GirolamoDistel, MartinRanieri, GirolamoDittmar, ThomasRaninga, PrahladDjavahery Mergny, MojganRanjan, AlokDoberentz, ElkeRanjan, KishuDobrescu, AmadeusRankovic, ZoranDobrovic, AlexanderRaoul, WilliamDobson, Tara H.W.Rapic, SaraDocea, Anca OanaRapozzi, ValentinaDodd, Rebecca D.Rascle, AnneDoescher, JohannesRashid, Mohammad HarunDogra, PrashantRasmussen, LeneDokic, IvanaRastegar, MojganDoksani, YlliRatajewski, MarcinDokudovskaya, SvetlanaRatnam, ManoharDoles, JasonRattan, RamandeepDolfini, DilettaRau, BeateDolznig, HelmutRaudenska, MartinaDome, BalazsRaugei, GiovanniDomenech, MaribellaRaut, SangramDomenici, GiacomoRavegnini, GloriaDomingo-Musibay, EvidioRavindranath, Aditi J.Domínguez-Pérez, DanyRavindranathan, SruthiDomínguez-Valentín, MevRawla, PrashanthDonadon, MatteoRay, ParthaDonati, BenedettaRay, SamriddhaDonati, ChiaraRay, SutapaDonati, MarcelloRaychaudhuri, PradipDong, JixinRazzokov, JamoliddinDong, PeixinReader, Jocelyn C.Donizetti, AldoRébé, CédricDonnelly, OliverRebelo, Sofia P.Donninger, HowardReclusa, PabloDonskov, FredeReddy, Pullagurala Venkata LaxmaDorffner, GeorgReddy, SakamuriDörrie, JanReddy, Sudhir PuttyDou, ZhixunRedell, MicheleDouglas, Steven D.Redmer, TorbenDovat, SinisaRedon, Christophe E.Dowty, JamesRedondo-Muñoz, JavierDoyle, Gerald V.Ree, Anne HansenDraghici, SorinReeh, MatthiasDragomir, MihneaRegazzo, DanielaDragon-Durey, Marie-agnesReich, Nancy C.Drakos, EliasReich, OlafDrapkin, RonnyReichner, JonathanDrutovic, DavidReichstein, David A.D’Souza, RoshanReigan, PhilipDu, WaReikvam, HåkonDu, XingrongReindl, JudithDubey, RaminReindl, JudithDubois, ChristopheReiner, Anne S.Dubois, LudwigReinheckel, ThomasDubrovska, AnnaReinisch, AndreasDucreux, SylvieReis, HenningDuda, DanRemaut, KatrienDudas, JozsefRemondelli, PaoloDuijvesz, DiederickRemotti, HelenDuina, Andrea A.Ren, BinDulong, SandrineRepasky, ElizabethDumaz, NicolasReszka, EdytaDumond, HeleneRety, StephaneDumont, FrédéricReuther, GaryDunn, IanReynisson FRSC, JóhannesDunne, RichardRezzani, RitaDurand, NishaRiabowol, KarlDussault, PatrickRiar, Amanjot KaurDutil, JulieRiaz, AhsunDutta, ArijitRibas, VicenteDutta, PinakiRicardo, SaraDutta, PranabanandaRiccardi, ClaudiaDutta, PranabanandaRiccardo, FedericaDwivedi, ChandraharRich, Jeremy N.Dziegiel, PiotrRichter, Sara N.Dzięgiel, PiotrRicordel, CharlesDziegielewska, BarbaraRiechelmann, HerbertDzierżanowski, TomaszRieckmann, ThorstenDzimitrowicz, AnnaRiegman, Peter H.J.Ear, JasonRiester, Scott M.Earl, JulieRiffelmacher, ThomasEarp, H. SheltonRigalli, Juan PabloEbert, Lisa M.Riggi, NicolòEbihara, LisaRimassa, LorenzaEboigbodin, KevinRinaldetti, SebastienEckert, FranziskaRinnerthaler, GabrielEckert, MarkRiobo, NataliaEckschlager, TomasRiond, JoëlleEder, Iris E.Riondino, SilviaEdhi, Muhammad MuzzammilRisbridger, GailEdmund Kim, EuishinRishi, ArunEdward Lawler, SeanRispoli, JosephEferl, RobertRitter, AndreasEfremov, Dimitar G.Rittman, TimothyEgland, Kristi A.Riva, PaolaEguchi, TakanoriRivero, FranciscoEibl, GuidoRivoltini, LiciaEid, NabilRizzo, FrancescaEigeliene, NataljaRizzolio, FlavioEisenstat, David D.Roa, JuanEisinger-Mathason, Tzipora S.KarinRoato, IlariaEl Bairi, KhalidRobe, Pierre A.Elas, MartynaRoberg, KarinElble, Randolph C.Roberts, Arthur G.El-Gewely, Mohamed RaafatRoberts, Megan C.Elisabeth, SmolleRobila, Valentina V.Elkabets, MosheRobles, AnaEl-Mallawany, Nader KimRoby, KatherineEl-Naggar, AdelRoca, XavierEmerling, BrookeRocchi, StéphaneEmilsson, LouiseRocha, SoniaEmmenegger, MarcRoche, Marc De LaEndersby, RaeleneRodeberg, DavidEndo, MakotoRoderburg, ChristophEndris, VolkerRodilla, VerónicaEnewold, Lindsey R.Rodolfo, CarloEngeland, ChristineRodolfo, MonicaEngeland, Christine E.Rodrigo, Juan PEnguita, FranciscoRodrigo, Juan P.Enjeti, AnoopRodrigues, DarioEnomoto, AtsushiRodrigues, Pedro MiguelEnomoto, MasaruRodrigues, RaquelEom, Gwang HyeonRodriguez Macias, Rocio IEpstein, Alan L.Rodriguez Peralvarez, ManuelErben, PhilippRodriguez, CristianEri, S. SrivatsanRodríguez, JavierEric Kast, RichardRodriguez-Flores, JuanErovic, Boban M.Rodríguez-García, CarmenEscargueil, AlexandreRodriguez-Vita, JuanEscolà-Gil, Joan CarlesRodríguez-Yoldi, María JesúsEscors, DavidRoedel, FranzEsmaeili Pourfarhangi, KamyarRoelink, HenkEso, YujiRogakou, EmmyEspel, EnricRoger, SebastienEspinosa, LluisRogister, BernardEspinosa-Diez, CristinaRojo, FedericoEsposito, FrancescoRojo, Maria AngelesEsposito, IreneRokavec, MatjazEsteve-Puig, RosauraRomano, AlessandraEstomba, Carlos Miguel ChiesaRomeo, GiovannaEsworthy, SteveRomero, CarmenEubank, TimothyRoncarati, DavideEudald, CasalsRoncucci, LucaEufemi, MargheritaRondon, AurélieEugenin, Eliseo A.Ronellenfitsch, UlrichEustace, Alex J.Rong, ChaoEvangelista, LauraRonquist, GunnarEvani, Shankar JaikishanRonsard, LaranceEvans, D. GarethRooman, IlseEvert, MatthiasRoos, Wynand PEvert, MatthiasRoque, AliciaEvinger, MarianRoque, NataliaEvrard, Solène M.Rosa, DivellaEykyn, ThomasRosado, JuanEyre, TobyRosa-Rosa, Juan ManuelEzzat, ShereenRose, April A.N.Ezzat, ShereenRose-John, StefanEzzat, ShereenRosell, RafaelFABBIAN, FABIORosenbaum, Jason N.Fabelo, HimarRosenzweig, AllisonFabris, LindaRosenzweig, DerekFaehling, MartinRösner, ThiesFagoonee, SharmilaRoss-Adams, HelenFahraeus, RobinRossi, RiccardoFaienza, FiorellaRossing, MariaFakhouri, Walid D.Roszczenko-Jasinska, PaulaFalasca, MarcoRotblat, BarakFalk, StephenRothschild, Sacha I.Falzone, LucaRoudbaraki, MoradFan, MeiyunRoue, GaelFan, ZhijinRouhi, ArefehFang, MingzhuRousseau, AudreyFang, XianjunRovida, ElisabettaFang, XiaolanRowther, Farjana B.Fang, Yi-PingRoxburgh, Campbell S.D.Fanzani, AlessandroRoy, SoumenFaraji, FarhoudRoyo, FelixFaraone Mennella, Maria RosariaRual, Jean-FrançoisFaraoni, IsabellaRuano, JuanFarge, GeraldineRubegni, PietroFarina, LorenzoRuberti, GiovinaFarina, NicholasRubina, KseniyaFarnham, PeggyRucci, NadiaFarolfi, AlbertoRuch, RandallFarr, Christine J.Rudd, SeanFassnacht, MartinRudd, SeanFavier, JudithRueda, BoFayette, JérômeRuess, Dietrich A.Fechner, HenryRuggiero, AntonioFedele, MonicaRui, HallgeirFederico, RavegliaRui, LixinFederico, Sara M.Ruiz Molina, DanielFeferman, TaliRuiz Pérez, María VictoriaFelicetti, FedericaRumney, Robin M. H.Fendt, Sarah-MariaRund, DeborahFeng, HuiRus, GuillermoFeng, YibinRusinek, DagmaraFeng, YingRussi, SabinoFenizia, FrancescaRusso, AntonioFennell, LochlanRusso, AntonioFeo, FrancescoRusso, DiegoFerbeyre, GerardoRusso, Matteo A.Ferguson, BrianRusso, SuzanneFernandes, RúbenRutgen, BarbaraFernández De Mattos, SilviaRyabchikova, Elena I.Fernandez, Hugo F.Rychahou, PiotrFernández-García, PaulaRyszawy, DamianFernández-Piqueras, JoséRzymowska, JolantaFernandez-Roldan, Jose AngelSaad, MohamedFernandez-Zapico, Martin E.Saba, NabilFerracini, RiccardoSaba, Nakhle S.Ferrante, PasqualeSabatier, RenaudFerrara, FelicettoSabol, MajaFerrara, Marianna LucianaSacchetti, BenedettoFerrari, DarisSachpekidis, ChristosFerrari, DarisSack Jr., George H.Ferrari, PaolaSack, UlrichFerraz Gonçalves, José AntónioSadasivam, MohanrajFerreira, Rui M.Saeed, KhalidFerrer, Valeria Pereira Saenko, VladimirFerri, Giovanni MariaSaez, CarmenFerrini, SilvanoSaga, KotaroFerriz-Martínez, Roberto AugustoSagrista, MariaFerroni, PatriziaSaha, AshirbaniFeske, StefanSaha, NirmalyaFeun, Lynn G.Sahni, SumitFiering, Steven N.Sahoo, SubhransuFilardo, EdwardSahu, Ravi P.Fillmore, HelenSai, KiranFilmus, JorgeSaid, NeveenFilson, Christopher P.Saida, SatoshiFinco, IsabellaSaikumar, PothanaFinegan, Katherine G.Saini, ShikhaFinocchiaro, GaetanoSaini, ShikhaFior, RitaSaini, ShikhaFiori, EnricoSaito, RyokoFiorillo, ClaudioSaitoh, ManabuFiorio Pla, AlessandraSakai, HiroyasuFishbein, LaurenSakai, ToshiyukiFisher, Jonathan S.Salani, RituFisher, Kerry D.Saleh, TareqFisher, PaulSalem, AbdelhakimFisher, ScottSalem, Joe-ElieFitzgerald, DavidSalemi, MicheleFiume, GiuseppeSaletti, VeronicaFiz, FrancescoSalih, Helmut RainerFlavell, RobertSalmon, Jessica M.Fleig, AndreaSalo, TuulaFleischer, Arthur C.Saltel, FrédéricFleitas, TaniaSalvatore, LisaFletcher, EliotSalvatore, RaffaFleury, FabriceSalzano, AndreaFliedner, StephanieSambandam, VaishnaviFlorea, Ana-MariaSambri, AndreaFogarty, GeraldSammartino, PaoloFoijer, FlorisSampathi, ShilpaFontanilles, MaximeSampurna, ChatterjeeForczmański, PawełSamson, AdelForest, FabienSamuel, TemesgenFornaguera, CristinaSamuels, BrianFornaguera, CristinaSanchez-Laorden, BertaForrester, HelenSánchez-Martínez, ConchaForster, JamesonSancho, PatriciaFort, PhilippeSancisi, ValentinaFortunato, AngeloSanda, TakaomiFortunato, OrazioSanderson, ChrisFosbøl, Marie ØbroSandhu, Nicole PunkayFoti, Pietro ValerioSandhu, Shahneen K.Foukakis, TheodorosSandstrom, JosefineFountzilas, ChristosSandulache, VladFracchiolla, Nicola StefanoSangiolo, DarioFrampton, GarrettSantarelli, AndreaFrancesca, LunardiSantofimia-Castaño, PatriciaFranceschi, FrancescoSantolla, Maria FrancescaFranceschini, DavideSantoni, GiorgioFrancipane, Maria GiovannaSantoni-Rugiu, EricFrancis, LewisSanz, AnaFrancisco, Ana PaulaSanz, Miguel A.Franco, Daiane G.Sapienza, CarmenFranco, LuisSapino, AnnaFranco, OmarSapozhnikov, Alexander M.Franco-Barraza, JanuszSaraiva, NunoFrancuz, TomaszSaretzki, GabrieleFranken, Nicolaas A.P.Sarhan, DhifafFranken, Nicolaas A.P.Sarkadi, BalázsFrassanito, AntonellaSarkar, DevanandFreeman, MichaelSarkaria, JannFrench, Pim J.Sarkozy, ClémentineFribley, AndrewSarma, SauravFrieboes, HermannSartini, MarinaFriedlander, TerenceSasaki, AtsuoFriedmann-Morvinski, DinorahSasaki, Atsuo T.Friedrich, ThomasSasaki, HirokiFriel, ClaireSastre, LeandroFrietze, SethSathe, Gajanan JalindarnathFrischauf, IreneSathekge, MikeFritah, SabrinaSato, HiromiFroehlich, LeopoldSato, KazuhideFröhlich, EleonoreSato, TakamiFu, WenjiangSatty, Alexandra.MFu, Xiao-AnSaule, SimonFu, YingSaunders, PhilippaFu, ZhengSavai, RajkumarFuereder, ThorstenSaviano, AntonioFujihara, HisakoSawyer, Sarah L.Fujii, Shin-ichiroSayans, Mario PerezFujii, TakumaSayar, HamidFujii, TomomiSaydam, OkayFujino, HiromichiScafoglio, ClaudioFujita, MasatoshiScalise, MariafrancescaFujita, MitsuguScanes, Colin G.Fujita, TetsujiScarpa, SusannaFujita, YukiScarpi, EmanuelaFujiwara, NaotoScavo, Maria PrincipiaFukumitsu, NobuyoshiSchadde, ErikFukunaga, TsukasaSchafer, ZacharyFukuoka, HidenoriSchaft, NielsFukushima, HiroshiSchank, Timo E.Fukushima, SatoshiSchapira, MatthieuFukushima, TsuyoshiSchartinger, VolkerFulciniti, FrancoSchatz, Jonathan H.Funaki, SoichiroScheidereit, ClausFunamizu, NaotakeScheinberg, David A.Funel, NiccolaSchenone, SilviaFüredi, AndrásSchepisi, GiuseppeFurler, Robert L.Schiavone, MarcoFurukawa, TatsuhikoSchiavone, NicolaFuruse, JunjiSchiffman, MarkFuruta, TakuyaSchildgen, VerenaFusco, AlfredoSchillaco, OrazioFusco, AlfredoSchirmer, BastianFusco, NicolaSchlaepfer, IsabelFushida, SachioSchlegel, JürgenGabr, MoustafaSchlesinger, MartinGabriel, EmmanuelSchlisio, SusanneGabrielli, BrianSchlom, JeffreyGagat, MaciejSchlosser, GittaGahete, ManuelSchmid, RafaelGaitskell, KeziaSchmidt, BarbaraGalardi, SilviaSchmidt, BarbaraGalas, SimonSchmidt, MarcGalasso, GiovanniSchmidt, Mirko HHGalata, ChristianSchmitt, Nicole C.Galderisi, UmbertoSchmitz, GerdGaleffi, FrancescaSchmitz, John C.Galetti, MariclaSchneider, BarbaraGaletti, MariclaSchneider, BjoernGalgani, MarioSchneider, GünterGalileo, Deni S.Schob, StefanGalizia, GennaroSchoeberlein, AndreinaGallardo, EnriqueSchofield, PaulGalle, PeterSchoormans, DounyaGallo, OresteSchrader, JörgGameiro, SofiaSchrader, Kasmintan A.Gan, Emily YipingSchrama, DavidGanapathy-Kanniappan, ShanmugasundaramSchraml, PeterGanau, MarioSchreck, Karisa C.Ganci, FedericaSchrefler, BernhardGandellini, PaoloSchuchert, Matthew J.Gandhi, Maher K.Schueler, JuliaGanesan, PalanivelSchuller, Hildegard M.Ganguly, AnutoshSchulz, Wolfgang A.Ganguly, AvishekSchulze, JohannesGanju, Ramesh K.Schulz-Heddergott, RamonaGao, DingchengSchumacher, UdoGao, JieSchürch, Christian M.Gao, MinSchwab, AlbrechtGao, ShuaiSchwaller, JuergGao, YangSchwartz, Robert E.Gao, YingSchwartzbaum, JudithGao, ZiyueSchwind, SebastianGarbers, ChristophSciumè, GiuseppeGarcia De Frutos, PabloSclafani, FrancescoGarcía-Escudero, RamónScorziello, AntonellaGarcia-Lora, Angel MiguelScott, AaronGarcia-Perez, Josselyn E.Scott, AndrewGarcia-Rico, EduardoScott, Christopher J.García-Vicente, Ana MaríaScumaci, DomenicaGarg, Abhishek D.Sean Hua, LimGarí, EloiSebastian, CarlosGariboldi, ManuelaSedgwick, Alanna E.Garinis, GeorgeSedic, MirelaGarlipp, BenjaminSeeboeck, RitaGarofalo, MariangelaSegatto, MarcoGarofalo, MariangelaSeidl, MaximilianGarrett, JoanSeidlits, StephanieGarrett, JoanSeino, ManabuGarrido, María JesusSeipel, KatjaGarvalov, Boyan K.Seki, NaohikoGąsior-Perczak, DanutaSellner, LeopoldGasparski, AlexanderSen, MalabikaGaston, KevinSengupta, DebrupGately, KathySeno, MasaharuGatselis, NikolaosSenter, Peter DGattei, ValterSeong, Ki MoonGaughan, LukeSerafin, ValentinaGaur, NishthaSergi, ConsolatoGautam, PrsonSério, SusanaGawel, DamianSerra, Antonio ArtiguesGazouli, MariaSerrano, DavideGazzaniga, PaolaSerrano, Maria JoseGe, XiaodongSerre-Beinier, VéroniqueGeary, SeanServais, StephaneGehl, Karen JulieServe, HubertGellert, LanSeshadri, MukundGener, PetraSethi, GautamGeng, FengSethi, GautamGentile, FabrizioSetty, BhuvanaGentile, FrancescoSeufferlein, ThomasGentile, SaverioSevcikova, SabinaGentle, Ian E.Sevenich, LisaGeorg, DietmarSeveri, StefanoGeorgakilas, AlexandrosSeverson, Aaron F.Georgakis, Georgios V.Seymour, John F.George Parsons, Kimberly SuzanneShah, TahirGeraci, FabianaShanmugam, MalaGerbino, AndreaShannon, KristenGericke, MartinShao, HaipengGerke, OkeShao, WeiGerling, MarcoShao, Yu-YunGertych, ArkadiuszSharabi, ShirleyGewirtz, David A.Sharan, ShyamGhayee, HansSharker, Shazid Md.Ghazanfari, LidaSharma, AnchalGhidini, MicheleSharma, ArishyaGhidini, MicheleSharma, GeetanjaliGhigna, ClaudiaSharma, ShwetaGhigo, AlessandraSharma, SudhaGhilli, MatteoShaul, Yoav D.Ghirga, FrancescaShaw, Adam C.Ghiringhelli, FrançoisSheikh, NadeemGhisoni, EleonoraSheldrake, HelenGhita, MihaelaSheltzer, JasonGhosh, SantoshShemanko, CarrieGhoshal-Gupta, SampaShen, Jia ZackGiacomelli, ChiaraShen, LishuangGiakoustidis, Dimitris E.Shen, Meng-RuGiammalva, Giuseppe RobertoShen, Tang-LongGiampieri, RiccardoShen, XiulongGianfredi, VincenzaShen, YuGiardino, AlessandroSheng, YueGibson, SpencerSheng, ZhiGilles, ChristineShenoy, NirajGiordano, AntonioShepherd, TrevorGiordano, AnttonioSherbet, Gajanan V.Giordano, CinziaSherman, JonathanGiorgi, Ugo DeSherman, MaraGiorgia, GurioliSheu, Gwo-TarngGiovannelli, PiaShewan, Louise G.Giovannetti, ElisaShi, JianGiovannetti, ElisaShi, LeiGiovannini, CatiaShiba, ShintaroGiovannucci, David R.Shibata, YasushiGiovarelli, MatteoShidal, ChrisGiovarelli, MirellaShieh, Dar-BinGirao, HenriqueShiga, KiyotoGirard, Pierre-MarieShigenori, NagaiGirbardt, ChristianShih, Chun-KuangGirnun, Geoffrey D.Shih, Jing-WenGiudice, ChiaraShikama, NaotoGiuliano, MichelaShim, JaejunGiuliano, Michela M.Shimaoka, MotomuGiulietti, AntonioShimizu, KazuoGiulietti, MatteoShimizu, MasahitoGius, DavidShimomura, HirofumiGiusti, IlariaShimono, YoheiGjini, EvisaShin, Vivian YvonneGlajchen, MyraShinmura, KazuyaGlare, Paul A.Shinohara, ShogoGlass, JonShintani, YasushiGlimelius, IngridShiota, SeijiGloor, BeatShiradkar, RakeshGnani, DanielaShirakami, YoheiGoda, TadahiroShmatov, MikhailGodlewska, MarlenaShnyder, SteveGoel, Hira LalShoji, TadahiroGoel, PeeyushShomura, MasakoGoel, Peeyush N.Shu, Chih-WenGoff, Stephanie L.Shukla, HemGoffin, VincentShukla, RuchiGogvadze, VladimirShukla, SiddharthGoins, William F.Shukla, SourabhGoldacre, Michael JShukla, SurendraGolding, JonShults, Elvira E.Goldsberry, Whitney N.Shun, Chia-TungGoldsmith, Jason R.Sibson, David RossGolubnitschaja, OlgaSiddharth, SumitGolubovskaya, VitaSiddiqui, ImranGomes-da-Silva, Lígia C.Sidhu, Stan B.Gómez De Cedrón, MartaSieber, FritzGómez, Pilar SánchezSieber, OliverGomez-Gutierrez, JorgeSiegelin, MarkusGonzales, Cara B.Sier, CornelisGonzález Granado, José MaríaSigala, SandraGonzález, ÁlvaroSigel, Carlie S.Gonzalez, DeyarinaSignore, MicheleGonzález, ItziarSikirzhytski, VitaliGonzalez, RogelioSikorski, AleksanderGonzález-Vallinas, MargaritaSill, HeinzGonzález-Vallinas, MargaritaSilva, Adriana RibeiroGood, PhillipSilva, SaraGoodier, Martin R.Silvennoinen, Olli JuhaniGoodman, KarenSilvestre, SamuelGoodwin, AnnabelSilvestri, IdaGordeeva, OlgaSilvestris, NicolaGordon-Weeks, AlexSima, LiviaGorgoulis, VassilisSimanainen, UllaGorini, FrancescaSimeonova, IvaGorlov, IvanSimmen, Frank A.Gornowicz, AgnieszkaSimmen, Rosalia C.M.Goroncy, AlexanderSimon, Michael S.Gorphe, PhilippeSimon, RonaldGorris, MarkSimonetti, GiorgiaGorska-Ponikowska, MagdalenaSimopoulou, MaraGorzelanny, ChristianSinger, Eric A.Gosciniak, GrazynaSingh, Ajay P.Goto, AkiteruSingh, AmritaGoto, TaichiroSingh, AnilGötze, KatharinaSingh, AnupGoubran, Hadi AlphonseSingh, BalrajGougelet, AngeliqueSingh, Brijesh KumarGough, DanielSingh, DeoGouilleux, FabriceSingh, Pankaj K.Govaere, OlivierSingh, Rakesh K.Gowda, NaganaSingh, RatnakarGozzini, AntonellaSingh, SarbjitGradilone, Sergio A.Singh, Sharda P.Graeber, Manuel B.Singh, Surya PratapGraf, NorbertSinghal, DhruvGraff, RebeccaSingleton, Dean C.Graham, Nicholas ASinha, DebottamGranados-Principal, SergioSioud, MouldyGranados-Principal, SergioSiozopoulou, VasilikiGranata, IlariaSisci, DiegoGranata, Vincenzasisley, KarinGrande, FedoraSisu, CristinaGrandemange, StéphanieSitcheran, RaquelGranucci, FrancescaSitcheran, RaquelGrassi, GabrieleSiu, Michelle K.Y.Grassilli, EmanuellaSiva, ShankarGratchev, AlexeiSivaguru, MayandiGraves, Edward E.Sivakumar, SushamaGray, Janet M.Sivaprakasam, SathishGray, Steven G.Siveke, JensGrazzini, GraziaSkendros, PanagiotisGrebien, FlorianSkorski, TomaszGreen, AlannaSlaney, ClareGreiner, JohannesSliwinska, AgnieszkaGreither, ThomasSlominski, AndrzejGreten, FlorianSmahel, MichalGreten, TimSmahel, MichalGrieco, DomenicoŚmieszek, AgnieszkaGriewank, Klaus G.Smilde, AgeGriffith, Thomas S.Sminia, PeterGriffith, Thomas S.Smith, Jill P.Griffiths, Ewen A.Smith, Steven S.Grigsby, Christopher L.Smolle, Maria AnnaGrimes, Mark L.Snyder, ChristopherGrimm, ChristinaSoares, HelenaGritli-Linde, AmelSobotta, ŁukaszGrösch, SabineSoda, HiroshiGross, GideonSöderberg-Naucler, CeciliaGrossi, FrancescoSohn, Eun-jungGrossi, ValentinaSokoloski, KevinGrossman, Ashley BarrySokolova, EvgeniyaGroult, HugoSolana, RafaelGrouzmann, EricSolanki, SumeetGrube, SusanneSolary, EricGrueneisen, JohannesSoleymani Abyaneh, HodaGrünewald, ThomasSolimando, Antonio G.Grunt, ThomasSolimando, Antonio GiovanniGrusch, MichaelSolinas, CinziaGruszka, Alicja MSoloff, Adam CGrzanka, MałgorzataSolovyeva, Valeriya V.Gu, FangyiSoltész, BeátaGu, WenyiSomasundaram, Dinesh BabuGu, YuchaoSomji, SeemaGuaita, SandraSomoza, ÁlvaroGuan, JikuiSomparn, PoorichayaGudey, Shyam KumarSon, Deok-SooGudey, Shyam KumarSonavane, ManojGudima, Severin O.Sonawane, AbhijeetGuenot, DominiqueSonda, SabrinaGuérin, FlorentSonego, MauraGuerlesquin, FrancoiseSong, Chang W.Gugel, IsabelSong, ChunhuaGugnoni, MilaSong, JaewhanGuha, Prajna P.Song, MinjungGuha, PrasunSong, SuGuidoboni, MassimoSong, XinhuaGuillerey, CamilleSong, YanGuirado, DamiánSonkin, DmitriyGuittaut, MichaëlSorber, LaureGulley, MargaretSorbye, Sveinung WergelandGumulec, JaromirSørensen, BritaGunaje, JayaramaSoria, FrancescoGunter, Jennifer H.Sorich, Michael JosephGuntinas-Lichius, OrlandoSorrell, J. MichaelGuo, FenghaiSorrenti, ValeriaGuo, SiqiSosna, JustynaGuo, WeiminSottnik, JosephGuo, XingyiSoubeyran, PhilippeGuo, YanSouglakos, JohnGuo, Zong ShengSounni, Nor EddineGupta, BrijSourbier, CaroleGupta, SanjaySouyri, MicheleGupta, SanjaySouza-Fonseca-Guimarães, FernandoGupta, SupritSoveral, GraçaGupta, VijayalaxmiSoverini, SimonaGurrapu, SreeharshaSpan, PaulGustav, StalhammarSpanakis, MariosGustavo, CallicoSpector, DavidGutschner, TonySpeirs, ValerieGuttery, David S.Sperti, CosimoHa, PatrickSpinella, MichaelHa, SanghoonSpinelli, LauraHaak, HarmSpitzer, DirkHaase, Astrid D.Spitzner, MelanieHackert, ThiloSprenger, CynthiaHackl, HubertSquillacioti, CaterinaHackshaw, AllanSredni, Simone TreigerHadoux, JulienSrinivasan, SwethaHaendler, BernardSrivastava, AkhilHagemann, Ian S.Srivastava, Pramod K.Hahne, JensSrivatsan, MalathiHalaban, RuthSrivenugopal, KalkunteHalbrook, ChristopherSroczynski, GabyHalkia, Evgenia A.Staaf, JohanHall, MarciaStack, SharonHallböök, FinnStack, SharonHallden, GunnelStacpoole, Peter W.Halmos, BalazsStadlbauer, AndreasHalmos, GáborStafford, Francis WilliamHaluza, DanielaStankevičius, VaidotasHam, Won-sikStanley, Jone A.Hamanishi, JunzoStark, Amy L.Hambloin, MichaelStark, Azadeh T.Hamburger, Anne W.Starostik, PetrHamidi, OksanaStarr, Timothy K.Hamieh, MohamadStaub, EikeHamilton, GerhardStaudacher, AlexanderHan, ChunhuiStauffer, PaulHan, DongStecca, BarbaraHan, Ki-HoSteel, Jason C.Han, Sun-YoungStefanini, LuciaHanash, Samir M.Stefano, CairoHancock, Wayne W.Steger, GertrudHanda, OsamuStehle, JörgHanenberg, HelmutSteinberg, FlorianHanker, AriellaStella, Giulia M.Hankinson, OliverStepanenko, Aleksei A.Hannafon, BethanyStepien, EwaHannah-Shmouni, FadyStepp, HerbertHans, GuyStevenson-Lerner, HeatherHansen, Adam E.Steward, RobertHao, JinsongStewart, Clinton F.Hao, MingangStewart, Lamonica V.Hao, YajingStewart, MurrayHao, YuhanStigliano, AntonioHarada, HisashiStone, Jon R.Harada, MamoruStope, Matthias B.Harada, MasakoStorti, PaolaHarashima, NanaeStossi, FabioHardingham, Jennifer E.Strauss, LauraHareendran, SangeethaStrickler, John H.Harikrishna, KommidiStrilic, BorisHarms, PaulStripp, Sven T.Harn, Horng-JyhStrisovsky, KvidoHarrell, J. ChuckStrnadel, JanHarris, Edward N.Ström, AndersHartley, AndrewStröm, LenaHartman, MariuszStruzik, JustynaHartmann-Johnsen, Olaf JohanStuani, LucilleHasegawa, MakotoStylli, Stanley S.Hasenoehrl, CarinaSu, Po-HsuanHassan, MohamedSu, Ruey-ChyiHassan, SaimaSu, RuiHasse, SybilleSu, Ying-HsiuHassel, Jessica CecileSuami, HirooHatakeyama, MasanoriSubramaniam, DharmalingamHatakeyama, ShingoSuda, KenichiHattori, NorimichiSuderman, MatthewHau, PeterSugarbaker, PaulHaugnes, Hege SagstuenSugimoto, MitsushigeHauser, PeterSugimoto, NaotoshiHavas, KristinaSugimura, HaruhikoHawkett, BrianSugiura, TsuyoshiHayashi, RyujiSujobert, PierreHayashi, TomokoSukhorukov, VladimirHaynes, JenniferSukocheva, OlgaHaynes, Nicole M.Sullivan, LucasHazane-Puch, FlorenceSumazin, PavelHe, AinaSun, Cheng-CaoHe, ChunboSun, DanHe, FengSun, GongqinHe, HongSun, JieHe, HongliangSun, KunHe, T.C.Sun, LiHeakal, YasserSun, QianHealey, GarethSun, WeiHeckman, Caroline ASun, WenyueHeegaard, SteffenSun, WujinHehlgans, StephanieSun, WujinHeikenwälder, MathiasSun, YapingHeiland, Dieter HenrikSun, YinHeinbockel, ThomasSun, ZhifuHeino, JyrkiSundahl, NoraHelland, ÅslaugSundaramoorthy, PasupathiHellstrand, KristofferSundararajan, RajiHelmerhorst, F.Sung, Wen-WeiHendriks, LizzaSupernat, AnnaHendrikx, Jeroen J.M.A.Suri, Jasjit S.Hendrix, DavidSuri, Jasjit S.Henen, Morkos A.Surov, AlexeyHeng, Henry H.Surov, AlexeyHenker, ChristianSuwan, KeittisakHenrique, RuiSuzuki, HiromiHenrique, RuiSuzuki, HiroyoshiHenry, Ryan A.Suzuki, MaikoHeo, Tae-HweSuzuki, MasatakaHerblot, SabineSuzuki, TakafumiHerbst, AndreasSvane, Inge MarieHerfindal, LarsSvanvik, JoarHerling, MarcoSwierczynski, JulianHermann, Patrick C.Swietach, PawelHernandez Rivas, Jesus MariaSyed, ViqarHernandez, AngelSykiotis, Gerasimos P.Hernández, ÁngelSymington, LorraineHerold, NikolasSyn, Wing-KinHerold-Mende, ChristelSzabò, IldikòHerr, Deron RaymondSzanto, MagdolnaHervouet, EricSzebeni, Gábor J.Herwig-Carl, Martina C.Szebeni, Gábor JánosHess, AngelaSzegezdi, EvaHess, Lisa M.Szeliga, JacekHesson, LukeSzeliga, MonikaHetta, Helal F.Szewczuk, Myron R.Heymann, DominiqueSzkandera, JoannaHiggins, Paul J.Szlosarek, Peter W.Hildreth, BlakeSznarkowska, AlicjaHildreth, BlakeSzutkowski, KosmaHiller, Jonathan G.Tabernero, ArantxaHiraga, ToruTacconelli, StefaniaHirano, HidekazuTada, ToshifumiHirata, KenjiTafani, MarcoHirosue, AkiyukiTafuri, AgostinoHiroyuki, ShibataTagliamonte, MariaHisada, YoheiTaguchi, AyumuHishikawa, YoshioTaillandier, DanielHo, Jar-YiTajima, TsuyoshiHo, VincentTakada, IchiroHo, Xuan-HungTakahashi, YutakaHo, Yuan-SoonTakakura, KazukiHoang-Vu, CuongTakamatsu, ShigeyukiHochrainer, KarinTakami, AkiyoshiHoeben, AnnTakeda, MasayukiHoehn, KyleTakeda, SenHoeppner, JensTakenaka, YukinoriHoffmann, MarkusTakeshita, FumitakaHoffmann, Michele J.Talbert, ErinHoffmeister, MichaelTalbot, ChristopherHohaus, StefanTallapaka, Shailendra B.Hojjat-Farsangi, MohammadTalukdar, Fazlur RahmanHojnik, NatašaTam, Wai LeongHoller, EggehardTamaian, RaduHollomon, Mario G.Tamori, AkihiroHölzel, MichaelTan, C. SabrinaHomma, Miwako KatoTan, Joanne T.M.Hong, In-SunTan, KarenHong, Ka L.Tanabe, ShihoriHong, Su-HyungTanaka, AkiraHooda, JagmohanTanaka, KozoHooda, JagmohanTanaka, NobuyukiHopkins, Ashley M.Tanaka, ShotaHori, ToshiyukiTanaka, TakujiHorsman, Michael RobertTanaka, YasuoHosen, NaokiTandra, Pavan KumarHoshiba, TakashiTang, AnnaHosokawa, YoichiroTang, DamuHossain, FokhrulTang, Ho LamHossain, Md KamalTang, Kai DunHosui, AtsushiTang, Kwan HoHour, Tzyh-ChyuanTang, YuanHouse, ImranTang, YuanHouston, Kevin D.Taniguchi, HiroakiHrstka, RomanTankiewicz-Kwedlo, AnnaHsaio, MichaelTantos, ÁgnesHshieh, Tammy TTao, ChenqiHsia, Shih-MinTao, JunyanHsiao, Hui-HuaTaphoorn, Martin J.B.Hsiao, Yi-HsuanTarallo, ValeriaHsieh, JamesTarantino, GiovanniHsieh, Pei-LingTarasova, Nadya I.Hsieh, Yi-HsienTaratula, OlenaHsu, Fei-TingTarnowski, MaciejHsu, Kuo-ShengTashima, ToshihikoHsu, Ping ChihTasso, RobertaHsu, Ren-JunTatebe, HisashiHsu, Sung-PoTateishi, KensukeHsu, Tsung-ITaura, KojiroHsu, TzuchiTauriello, DanieleHsu, Ya-LingTauriello, DanieleHsu, Yi-ChiungTauro, MarilenaHu, BaoliTavares, AnthonyHu, BingTavassoli, MahvashHu, GuangzhenTaylor, AlexandraHu, MinTaylor, SamuelHu, TaoboTazawa, HiroshiHuang, Chao-ChengTchoghandjian, AurelieHuang, Chin-ShiuTe Riele, HeinHuang, Guan-JhongTee, Andrew R.Huang, HaoTeh, Muy-TeckHuang, Jason H.Teicher, Beverly A.Huang, Ruea-YeaTeijeira, ÁlvaroHuang, Shu-PinTeixeira, Ana LuísaHuang, Tse-HungTeixidó, CristinaHuang, Tse-HungTel, JurjenHuang, Tze-SingTella, Sri HarshaHuang, Weei-YuarnTelleria, Carlos M.Huang, Wei-ChienTellier, MichaelHUANG, Wen-ChinTemblador, ArturoHuang, XiTempleton, Arnoud JHuang, XiaohuTempst, PaulHuber, StephanTen Hagen, Timo L. M.Hudlikar, Rasika R.Tenbusch, MatthiasHuen, AurisTeng, NelsonHughes, Daniel C.Teng, YongHui, Kam ManTennstedt, PierreHui, Kwai FungTeodoro, Anderson JungerHumbert, MagaliTer Heine, RobHumphries, BrockTerai, MizueHung, Jan-jongTerai, ShujiHung, Ming-SzuTermolino, PasqualeHung, Shih-KaiTerracciano, DanielaHuppi, KonradTerradot, LaurentHuppi, KonradTerranova, ChristopherHur, HoonTerzolo, MassimoHussain, Ahmad FawziTesta, UgoHussaini, HaizalTevosian, Sergei G.Hussein, SarfarazTezil, TugsanHussey, JulietteThaker, Youg RajHusson, OlgaThakor, NehalHutchison, Robert E.Thakur, Sunitha N.Hyde, Ricia KatherineThaxton, JessicaHyun, Dong ChoonTheodoropoulos, GeorgeHyun, Kyung-AThevenod, FrankIaccarino, IngramThiel, KristinaIacobazzi, VitoThiery-Vuillemin, AntoineIadevaia, ValentinaThomas, ClémentIbrahim, Sherif AbdelazizThomas, Seddon Y.Idbaih, AhmedThomas, T.J.Ido, YasuoThomas, WilliamIdogawa, MasashiThomas, XavierIdris, AdiThoms, JohnIeni, AntonioThomson, TimothyIentile, RiccardoThrower, EdwinIgawa, SatoshiThuringer, DominiqueIgaz, PeterThuzar, MoeIkeda, FusaoTiao, Greg M.Ikura, YoshihiroTiberio, Guido Alberto MassimoIliakis, GeorgeTibullo, DanieleIllert, LenaTicha, IvanaIm, Chang-NimTiffen, Jessamy C.Imai, YoichiTimchenko, NikolaiImamura, MasahiroTimmers, HenriImbriano, CarolTimoshenko, AlexanderImperlini, EstherTinhofer-Keilholz, IngeborgInga, AlbertoTipping, JillIngallina, CinziaTiribelli, ClaudioInoue, KazushiTiriveedhi, VenkataswarupInoue, SatoshiTirosh, AmitInoue, TakahiroTischler, ArthurIorio, EgidioTiso, NatasciaIppoliti, RodolfoTitus, MarkIppolito, LuigiTixeira, RochelleIrato, PaolaTobias, GerardIriuchishima, HironoToedebusch, ChristineIrshad, SheebaToffoli, GiuseppeIrum, KhanTogo, ShinsakuIsella, ClaudioToh, Tan BoonIshida, FumihiroToietta, GabrieleIshikawa, KenjiToillon, Robert-AlainIshizuka, TakumiTokas, TheodorosIsidoro, CiroTokino, TakashiIsokpehi, RaphaelTolomelli, AlessandraIto, Ken-ichiTomaić, VjekoslavIto, ShuheiTomasetti, MarcoItoh, KazuyukiTomassetti, AntonellaItou, JunjiTomita, YusukeItuarte, PhilipTomiyama, ArataIurescia, SandraTommasi, StefaniaIwamoto, ShotaroTommasino, FrancescoIwamura, HiroyukiTomuleasa, CiprianIwasawa, ShunichiroTonetti, Debra A.Iyengar, Prasanna VasudevanTong, JessicaIzumi, KojiTopala, IonutIzzi, ValerioTorigoe, KanjiroIzzo, PaolaTorigoe, ToshihiroJaakkola, Panu M.Torka, RobertJacenik, DamianTorng, Pao-LingJacob, JosephTorrado, EgídioJacobs, Aaron T.Torre, MarialuisaJacobs, JulieTorres-Ayuso, PedroJacomin, Anne-ClaireTorti, MauroJager, Martine JToruner, Gokce AltayJaguva Vasudevan, Ananda AyyappanTosato, GiovannaJahani-Asl, ArezuToshimitsu, YamaokaJain, VaibhavTot, TiborJaing, Tang HerToth, ZsoltJalkanen, SirpaTóth-Molnár, EditJan, LilyTougeron, DavidJanakiraman, HarinarayananTouzé, AntoineJanczar, SzymonTovoli, FrancescoJanecki, MarcinTovoli, FrancescoJang, Seung HunTowers, ChristinaJang, Sung-WukToyomasu, YoshitakaJanga, MadhusudhanaTozer, GillianJanic, AnaTran, Anh NhatJaniszewska, MichalinaTran, Anh NhatJankovic, MomciloTreglia, GiorgioJansen, GerritTrenti, TommasoJanuchowski, RadosławTreps, LucasJanuchowski, RadosławTrilling, MirkoJardin, IsaacTrilling, MirkoJares, PedroTrindade, AlexandreJargin, Sergei V.Tripathi, ManishJarstfer, Michael BruceTripathi, ManishaJaskula-Sztul, RenataTrivedi, RachanaJászai, JózsefTrobaugh-lotrario, A.D.Jat, Parmjit S.Trobaugh-Lotrario, Angela D.Jauberteau, Marie-OdileTrommer, MaikeJavaheri, TaherehTrpkov, KirilJaworska, DagmaraTruffi, MartaJayaprakash, PriyamvadaTruong, DanhJazirehi, Ali R.Tryndyak, VolodymyrJean-Francois, FrantzTsai, Chia-Hung DylanJeck, William R.Tsai, Chieh-ChihJégo, GaëtanTsai, Jen-PiJenkins, RussellTsai, KelvinJensen, Lars HenrikTsai, Ming-ShaoJeon, JonghoTsai, Shih-changJeon, Yoon KyungTsai, Wan-ChiJeong, JoonTsai, Wen SyJeong, Keun-YeongTsai, Yuan-ChenJeong, Seung MinTsang, Allen W.Jeong, YangsikTsang, Kwong YokJernberg-Wiklund, HelenaTsang, Suk-YingJeroen T, BuijsTsapogas, PanagiotisJeronimo, CarmenTschernig, ThomasJerónimo, CarmenTseng, Ruo-ChiaJewett, AnahidTsiaoussis, JohnJežek, JanTsigelny, Igor F.Ji, JianguangTsigos, ConstantineJi, PengTsilimigras, Diamantis I.Jia, DongyaTsirigotis, PanagiotisJiang, JianxiongTsoi, HoJiang, JingruiTsolakis, Apostolos V.Jiang, Shih ShengTsoulfas, GeorgiosJimi, EijiroTsuchiya, HiroyukiJin, ChunyuanTsuchiya, KaoruJin, GeTsuchiya, TomoshiJin, JiefuTsuchiya, YuichiJin, XinTsui, Kuan HaoJing, RanTsuji, TakaoJirkovská, AnnaTsukamoto, HirotakeJochum, ChristophTsumura, HideyasuJoffe, ErelTsunematsu, TakaakiJohansson, PeterTsushima, TakahiroJohns, TerranceTu, Benjamin P.Johns, Terrance G.Tu, Shih-HsinJohnson, Daniel E.Tu, ThomasJohnson, David G.Tubaro, CristinaJohnson, Peter FTuca, AlbertJolly, M.K.Tucci, MarcoJonckheere, NicolasTulasne, DavidJoosten, BertTuma, PamelaJordan, AndreasTuna, MusaffeJordan, PeterTunaru, SorinJordanova, Ekaterina S.Tuohy, VincentJørgensen, Anders PalmstrømTurato, CristianJosefsson, AndreasTurcan, ŞevinJoseph, ShannonTurchetti, DanielaJoshi, Bharat H.Turdo, AliceJoshi, KamalTuretta, MatteoJoshua, Anthony M.Turley, Eva A.Josse, ClaireTurner, SunnaneJoubert, NicolasTurunen, JoniJoussen, Antonia M.Tuyaerts, SandraJu, XinshengTyagi, PradeepJudge, AndrewTzanakakis, George N.Juhlin, ChristoferTzankov, AlexandarJuif, Pierre EricUccello, MarioJung, Alain C.UCHIDA, KunitoshiJung, Chan KwonUetake, HiroyukiJung, GuhungUlasov, IlyaJung, KlausUlf, KahlertJunker, KerstinUlivi, PaolaJustin L., BrownUlivi, PaolaKaanders, HansUmemura, AkiraKaczmarek, ŁukaszUmemura, AtsushiKaesmann, LukasUmemura, NaokiKahlert, ChristophUmezawa, MasakazuKahn, MichaelUmmarino, SimoneKaido, ToshimiUngefroren, HendrikKaifi, JussufUngerstedt, Johanna S.Kajimoto, ShinjiUnseld, MatthiasKakar, ShamUpadhyay, GeetaKakar, Sham S.Upadhyay, KiranKakeji, YoshihiroUpadhyaya, JasbirKakkassery, VinodhUpham, Brad L.Kalafatis, MichaelUpham, Brad L.Kalamvoki, MariaUprety, DipeshKalayda, GannaUrabe, YujiKaldis, PhilippUrbanelli, LorenaKalinin, AlexandrUrbanelli, LorenaKalish, Jennifer M.Urbanska, Edyta MariaKalita-de Croft, PriyakshiUrbanucci, AlfonsoKallergi, GalateaUrick, Mary EllenKalpadakis, ChristinaUshmorov, AlexeyKalsi, TaniaUskoković, VukKaltschmidt, ChristianUslu, UgurKamai, TakaoUsui, TatsuyaKamath, DivyaUsui, YoshihikoKamato, DanielleUziel, OritKambhampati, Siva PramodhVacante, MarcoKamimura, KenyaVachtenheim, JiriKamp, MarcelVáclavíková, RadkaKanakkanthara, ArunVaidya, AmitaKanda, TatsuoVaiman, DanielKanda, TeruVainio, SeppoKandil, SaharVakili, KhashayarKandimalla, RajuValable, SamuelKane, EleanorValdmanis, PaulKanemitsu, YukihideValente, SabrinaKang, ChenValentine, Rudy J.Kang, Hong-YoValentovic, MonicaKang, Hyun-CheolValenzuela, RodrigoKang, JeonghyunVallée, AlexandreKang, Koo JeongVallet, SoniaKang, WeiValli, De ReKango-Singh, MadhuriVallone, DanielaKanno, AtsushiValter-Kocsi, KrysztinaKant, ShivaValtorta, SilviaKao, Shu-HueiValverde, ClaudiaKapetanaki, MariaValverde, Jose R.Kappler, RolandVan Bergen En Henegouwen, Paul M.P.Kapral, MA£GORZATAVan Dam, PeterKapur, NeerajVan De Stolpe, AnjaKar, AdwitiyaVan De Vijver, KoenKar, AdwitiyaVan den Bergh, HubertKar, AnirbanVan Den Beuken-van Everdingen, Marieke H.J.Karakoula, KatherineVan den Brekel, Michiel Wilhelmus MariaKaramouzis, MichalisVan Der Kogel, Albert J.Karayiannakis, AnastasiosVan Der Lugt, JasperKarmakar, ParthaVan Der Schaaf, ArjenKarmazyn, MorrisVan Dijk, Elon H. C.Karp, JudyVan Gent, DikKARPIŃSKI, TOMASZ MVan Gompel, Jamie J.Karstedt, Silvia VonVan Gool, Stefaan W.Kasai, FumioVan Laarhoven, Hanneke W.M.Kasenda, BenjaminVan Luijk, PeterKashaninejad, NavidVan Maaren, Marissa C.Kashiwagi, ShinichiroVan Meerten, TomKashofer, KarlVan Meeteren, Laurens A.Kashuba, ElenaVan Noorden, RonKasinski, AndreaVan Rechem, CapucineKasprzak, AldonaVan Rhoon, Gerard C.Kastrati, IridaVan Seuningen, IsabelleKataoka, HiromiVan Tine, BrianKatapodi, Maria C.Van Tine, Brian AndrewKathawala, RishilVanan, Magimairajan IssaiKathuria, HimanshuVanBrocklin, MatthewKatira, ParagVanderhyden, BarbaraKato, Takamitsu AVandyke, KateKato, Takamitsu A.Vankelecom, HugoKato, YasumasaVaqué, José PedroKatoh, HironoriVaquero, JavierKatrinli, SeymaVara, DinaKatseli, AnastasiaVaras-Godoy, ManuelKatsila, TheodoraVarga, GergelyKatuchova, JanaVarki, Nissi M.Katz-Brull, RachelVaron, ChristineKaufman, BrettVasicek, OndrejKaul, RashmiVasko, VasylKaur, AmanpreetVassiliadis, ThemistoklisKaushik, Nagendra KumarVassilopoulos, GeorgeKawamura, TakahisaVasudevan, Sanjeev A.Kawano, KouichiroVathipadiekal, VinodKawashima, HirotoVåtsveen, TheaKawauchi, KeikoVaupel, PeterKawauchi, KeikoVäyrynen, Juha P.Kawiak, AnnaVazquez, EstherKazanietz, MarceloVeeramani, SureshKe, Po-YuanVeeravalli, VijayabhaskarKe, YouqiangVelasco, EladioKeenan, JoanneVelegzhaninov, Ilya O.Keightley, CristinaVelikova, TsvetelinaKeijzers, GuidoVelpula, KiranKelada, Olivia J.Veltkamp, ChristianKelley, EricVenerando, AndreaKello, MartinVenkatadri, RajkumarKelly, Gemma L.Venkatadri, RajkumarKelly, William K.Venkatesan, ThiagarajanKemp, Michael G.Venneti, SriramKennedy, Michael A.Venters, BryanKenner, LukasVentoso, IvánKenyon, JulianVenturella, RobertaKepley, ChristopherVenza, MarioKern, PeterVerde, GaetanoKerr, IanVerdier, MireilleKeshamouni, Venkateshwar G.Verdijk, Robert M.Kesharwani, SiddharthVergara, DanieleKessler, SonjaVerghese, ShilpiKettenbach, JoachimVerma, Navin KumarKeung, Albert J.Verma, PriyankaKeyaerts, MarleenVermeer, Paola D.Khadka, Vedbar SinghVersteeg, Anne L.Khaled, NouraVessoni, AlexandreKhalilia, Walid MahmoudVetrie, DavidKhammanivong, AliVetter, MarcusKhan, AkbarViapiano, MarianoKhan, Mohammad AslamVibhute, SandipKhanim, FarhatVicentini, CaterinaKhatri, BhuwanVideira, Romeu A.Kholová, IvanaVidili, GianpaoloKhoo, Bee LuanViel, SébastienKhoo, Ui-SoonVilaseca, IsabelKhoury, Joseph D.Villa Morales, MaríaKhurshed, MohammedVilla, StefaniaKhushi, MatloobVillalobos, CarlosKibriya, MuhammedVillalonga, PriamKidane, DawitVilla-Morales, MaríaKieffer, BrunoVillani, RosannaKiemer, AlexandraVillarini, AnnaKiilgaard, Jens FolkeVillegas-Sepulveda, NicolasKikuchi, HidehikoVinall, RuthKilic, EmineVinciguerra, ManlioKim, BohkyungVinciguerra, ManlioKim, BongleeVinkemeier, UweKim, Byung SooVinken, MathieuKim, DaeViola, GiampietroKim, Hyo SongVisani, GiuseppeKim, Hyung SikVisone, RosaKim, HyungjinVisser, LydiaKim, Jae-HoonVitale, AlessandroKim, Jae-SungVitale, MassimoKim, Jin-WookVitale, Salvatore GiovanniKim, Jung HanVitorino, RuiKim, KwangheeVivacqua, AdeleKim, Nam DeukVivas Mejia, PabloKim, Raymond HVivo, MariaKim, Raymond HVizeacoumar, FrancoKim, Ryong NamVlajnic, TatjanaKim, RyungsaVolarevic, VladislavKim, Sang WunVoliani, ValerioKim, Sang-WeVolinia, StefanoKim, Seok JinVon Amsberg, GunhildKim, So YeonVon Bubnhoff, NikolasKim, Sung-HoonVon Der Thüsen, JanKim, SuwonVona-Davis, LindaKim, Tae HyunVora, MehulKim, Tae IlVotta-Velis, GinaKim, TaewanVoutsadakis, IoannisKim, Yong-miVoutsinas, Gerassimos E.Kim, Yu ChulWackernagel, WernerKim, Yu YeongWada, Ken-IchiKim, Yun HakWada, RyuichiKim, Yun-HeeWade, Mark A.Kimpel, JanineWadehra, MadhuriKimura, KiminoriWadhwa, RenuKimura, MasahiroWahl, Daniel R.Kinjo, TakaoWahlestedt, ClaesKinoshita, TakayoshiWakatsuki, MasaruKinoshita, TomonariWałajtys-Rode, ElżbietaKinugasa, HideakiWalch, AxelKiontke, AndreasWald, OriKirienko, MargaritaWali, AnilKirken, Robert A.Walinder, RobertKischel, PhilippeWallace, Nicholas A.Kiss, RobertWalline, HeatherKitatani, KazuyukiWalsh, Scott T.R.Klampatsa, AsteroWalters, ZoeKlampfer, LidijaWalther, WolfgangKlapdor, RüdigerWan, YuanKleeff, JörgWandosell, FranciscoKleibl, ZdeněkWang, Bang-AnKlein, AndreasWang, Chi ChiuKlein, BrittaWang, ChihueiKlein, HannahWang, Ching-ChiungKlein, UlfWang, ChunKlijanienko, JerzyWang, DaifengKlimczak, AleksandraWang, DennisKlughammer, JohannaWang, EdwinKlussmann, Jens PeterWang, Fu-NienKlymenko, YuliyaWang, GuankuiKnappe, UlrichWang, GuocanKnittelfelder, OskarWang, HsiuyingKnösel, ThomasWang, Jaw-YuanKo, Jiunn- LiangWang, Jaw-YuanKobayashi, HiroakiWang, JiongweiKobayashi, TetsuoWang, JueKöbel, MartinWang, KaiKöberle, BeateWang, KaiKobold, SebastianWang, KepengKocer, ArmaganWang, Lai-XiKoch, AlexanderWang, LiangKochan, Grazyna T.Wang, LihuiKodama, TakahiroWang, LizhongKodet, OndrejWang, Po-HuiKoelsche, ChristianWang, RanranKoff, Jean L.Wang, RuiKoga, FumitakaWang, RuoxiangKoga, YoshikatsuWang, ShueKohanbash, GaryWang, ShyiwuKöhl, GudrunWang, Tong-HongKohn, BrendaWang, Tong-HongKoizume, ShiroWang, Wei-ShuKoizumi, MitsuruWang, WenqiKojima, FumiyoshiWang, XinwenKojima, TakashiWang, XueKok, H.P.Wang, XueKok, Henny Petra.Wang, Yi-zhiKok, Victor C.Wang, YulingKokabu, ShoichiroWang, ZhishanKolettas, EvangelosWanibuchi, HidekiKolling, StefanWanis, Kerollos N.Kolot, MikhailWaring, PaulKolota, AleksandraWarnakulasuriya, SamanKomohara, YoshihiroWas, HalinaKonantz, MartinaWasilewski, AndreaKondapalli, ChandanaWasylishen, AmandaKondo, MotonariWatabe, TadashiKondo, TsunenoriWatson, PhilipKondylis, VangelisWatterson, D. MartinKoneru, BhuvaneswariWaugh, Mark G.Kong, Doo-SikWawruszak, AnnaKong, Michael G.Weber, Georg F.Kong, Sun YoungWeber, GüntherKonig, HeikoWeber, MichaelKonishi, HirotakaWebster, MarieKonishi, MasaakiWebster, Marie R.Konkol, YvonneWee, JosephKoo, Bon SeokWege, Anja KathrinKoo, Hee-beomWei, ChangyongKoo, Ja SeungWei, David ChangliKopaczyńska, MartaWei, Hong-JianKopanja, DraganaWei, Jun S.Kopechek, JonathanWei, ShuanzengKorin, NetanelWei, Wei-ChyouKorsching, EberhardWei, WenyiKortylewski, MarcinWei, YonglongKoshiyama, MasafumiWeidenhofer, JudithKosinsky, Robyn LauraWeigand, Julia E.Kota, SatyaWeigel, Ronald J.Koul, HariWeighardt, HeikeKoupenova, MilkaWeinberg, FrankKourkoumelis, NikolaosWeinberg, RobertKoutelou, EvangeliaWeinfeld, MichaelKoval, AlexeyWeinstein, JuliaKowalczyk, Anne E.Weisberg, EllenKowalska, AldonaWeiser, Daniel A.Koyama, ShoheiWelen, KarinKoyama, YuWellner, Ulrich FriedrichKozlowski, PiotrWelsh, MichaelKrämer, Oliver H.Welshhans, KristyKranz, MathiasWempe, MichaelKratochwil, ClemensWen, WeiKrause, GünterWen, Yu-ChingKreeger, PamelaWeng, ChingfengKreis, Nina-NaomiWernig, FlorianKrenács, TiborWesseling, PieterKretz, Anna LauraWesterhausen, ChristophKriebardis, Anastasios G.Westermarck, JukkaKrieg, AndreasWesthaus, SandraKrishnan, Viswanathan V.Westhoff, Mike-AndrewKroeze, Stephanie G.C.Westover, KennethKrstic, JelenaWeyemi, UrbainKrug, SebastianWharen, Robert E.Krug, SebastianWhelan, Kelly A.Krüger, MarcusWhite, PeterKrum, Susan A.Whitehall, VickiKruspig, BjornWhitman, GaryKrzyżek, PawełWibowo, DavidKshirsagar, PrakashWicha, MaxKsiążek, KrzysztofWicki, AndreasKu, Cheol-ryongWickström, MalinKuban-Jankowska, AlicjaWieczorek, EdytaKuchenbauer, FlorianWiemer, Erik A.C.Kucheryavykh, Lilia Y.Wieser, RotraudKudela, ErikWietrzyk, JoannaKudo, YasuseiWiggermann, PhilippKuiper, RolandWight, ThomasKukuruzinska, Maria A.Wikström, PernillaKulangara, KarinaWilczek, BrigitteKulasinghe, AruthaWilley, Christopher D.Kulawiak, BoguszWilliams, Brent A.Kulcenty, KatarzynaWilliams, BrianKulik, GeorgeWilliams, CeciliaKulkarni, PriyankaWilliams, OwenKumar Patel, SravanWilliams, RyanKumar, AkhileshWilliams, Stephen J.Kumar, AshokWilm, MatthiasKumar, GauravWilmink, HannekeKumar, KrishanWilting, JorgKumar, NareshWiner, IraKumar, SuneelWingert, RebeccaKumar, SunilWinkler, JohannesKumar, SushilWinter, AlexanderKume, TsutomuWirsching, Hans-GeorgKumi-Diaka, JamesWirth, MarkusKunderfranco, PaoloWirth, ThomasKundu, AishwaryaWischhusen, JörgKuno, HirofumiWise-Draper, Trisha M.Kuno, ToshiyaWitcher, MichaelKunoh, TatsukiWittmann, JürgenKunz, MeikWitzel, IsabellKuo, Chao-HungWohl, StefanieKuo, Po-LinWojtas, BartoszKuo, Sung-HsinWojtukiewicz, MarekKurenova, Elena V.Wolf, ElmarKurokawa, ManabuWong, DavidKurokawa, MineoWong, Hovy Ho-WaiKurth, InaWong, Kelvin K.Kuryk, LukaszWong, Pamela T.Kuryk, LukaszWong, Richard W.Kuryłowicz, AlinaWong, Stephen T. C.Kusaczuk, MagdalenaWong, Sze Chuen CesarKusmartsev, Sergei A.Woo, ShiaoKuwahara, MasayasuWoo, So-YounKuźnik, NikodemWozniak, Ann L.Kuzuya, TeijiWright, CatherineKveberg, LiseWu, Alexander THKwok, Hang Fai (Henry)Wu, CenKwon, Hyung JooWu, Chiao-EnKwon, Jae SungWu, Chin-ChungKwon, Seong YoungWu, Chun-ChiKwong, JosephWu, ChungChunKwong, Lawrence N.Wu, Chung-ChunKyprianou, NatashaWu, ErxiLa Rocca, Renato V.Wu, ErxiLa Rosa, Valentina LuciaWu, I-ChenLaboisse, Christian L.Wu, JiandeLaganà, Antonio SimoneWu, Mei-YiLahiri, TanayaWu, Meng-YuLahooti, HooshangWu, Ting-FengLai, CatherineWu, Tzong-YuanLai, Chih-HoWu, Yuan HuaLai, James J.Wurdak, HeikoLai, KeaneWykes, MichelleLai, Ming-DergXi, GuifaLai, QuirinoXia, BingLai, YandongXia, JunLaird, DaleXia, JunfeiLakshmikuttyamma, AshakumaryXiang, Shi-huaLallemand, FraņcoisXiang, XinLalli, EnzoXie, HongLalloué, FabriceXie, QianLam, Kim-HungXie, XiaopingLamango, Nazarius S.Xie, ZhiqunLamar, John M.Xing, FeiLambert, PaulXiong, ZhaohuiLambertini, MatteoXu, DazhongLandazuri, NataliaXu, HongyanLandreville, SolangeXu, WanpingLandry, JosephXu, WenyanLandström, MareneXu, YLang, RolandXu, YanLangelaan, David N.Xu, YuexinLanger, RupertXu, YungangLangsenlehner, TanjaXue, XiangLanguino, LuciaYadavalli, TejabhiramLannin, Donald R.Yaddanapudi, KavithaLao, ChunhuanYagüe, ErnestoLapidus, Rena S.Yakirevich, EvgenyLappano, RosamariaYakovlev, VasilyLapucci, AndreaYallapu, MuraliLara-Otero, KarlenaYamada, TaketoLardone, Patricia J.Yamada, TerumasaLarijani, ManiYamada, YoheiLarionov, VladimirYamaguchi, KenLaRocque, JanYamaguchi, RyujiLarribère, LionelYamakuchi, MunekazuLarsen, Anders ChristianYamamoto, YusukeLasfar, AhmedYamamura, SoichiroLasfar, AhmedYamanaka, RyuyaLasseigne, Brittany N.Yamanishi, KiyofumiLaterra, JohnYamaoka, ToshimitsuLathia, JustinYamasaki, TakahiroLattanzio, RossanoYamashita, HirokoLaudisi, FedericaYamauchi, AkiraLauricella, MariannaYan, BowenLaurino, SimonaYan, Catherine T.Lauriola, MattiaYan, ChunhongLausch, AnthonyYan, DayunLauth, MatthiasYan, DongqingLauth, MatthiasYan, JunLautz, Timothy B.Yan, ShanLavdaniti, MariaYanda, MuraliLaw, Man FaiYang, Chih-JenŁawicki, Sławomir ł.Yang, DaQingLawlor, Elizabeth RYang, GabsikLazarević, VladimirYang, HaifengLazaridis, GeorgiosYang, Hung-WeiLe Romancer, MurielYang, Jer-YenLe, MinhYang, Jiann-JouLe, Quynh-ThuYang, Jyh-ChinLeal, Ana MendesYang, LiliLebaron, RichardYang, LiuLechel, AndreYang, Pei-MingLeclercq, IsabelleYang, Rong-SenLee, A.-W.Yang, Shun-FaLee, ByungheonYang, WancaiLee, Chang-HoonYang, Wei-hsiungLee, Che-HsinYang, XiaomingLee, Cheng-IYang, YidongLee, Chia-HueiYang, YongkangLee, Chia-HwaYang, Yueh-Hsun KevinLee, Chien-KuoYang, ZengquanLee, Chong-KilYano, HajimeLee, ChuheeYao, Chao-LingLee, Dae-HeeYao, JianLee, Delphine J.Yao, JiayiLee, GyudoYap, Celestial T.Lee, Hsin-ChenYasuhara, TakaakiLee, HsinyuYatkin, EmrahLee, Hsueh-TeYe, KeqiangLee, HueiYe, LinLee, Hwa-YongYe, WenleiLee, Jae-HoYe, YanqiLee, JandeeYe, YunpengLee, Je-jungYe, ZhongLee, Jeong-HoonYee, DouglasLee, JieYeh, Chi-TaiLee, JiyunYeh, Chun-NanLee, Jun HeeYeh, Kun-TuLee, Keun SeokYeh, Shu-LanLee, MariaYeo, SynLee, Ming-ShyueYeung, Kam C.Lee, Moo-SeungYeung, Tsz-LunLee, NormanYi, ZongbiLee, Sang HunYilmaz, MusaLee, Sang-HanYing, HaoqiangLee, So YeongYing, MichaelLee, SookyungYlostalo, Joni H.Lee, Tae SupYochum, Gregory S.Lee, Wei-HwaYohe, MarielleLee, Wen-HwaYohe, Marielle E.Lee, ZhenghongYokobori, TakehikoLeemans, KathleenYokoi, KenjiLeenders, William P.J.Yokota, KenjiLegembre, PatrickYoneda, ToshiyukiLegendre, FlorenceYonesaka, KimioLeggatt, GrahamYoo, ChanghoonLegler, Daniel F.Yoo, Ji YoungLehen’kyi, V’yacheslavYoo, So YoungLehmann, Brian D.Yoo, So YoungLehnhardt, MarcusYoo, Young-ChulLehti, KaisaYoo, Young-DoLeiphrakpam, PremilaYoon, Do-YoungLejniece, SandraYoon, Hyeun JoongLemanska, AgnieszkaYoon, IlLemieux, KiTaniYoon, Kyong-AhLemke, JohannesYoon, Sung-SooLemma, SilviaYoon, SunochLemonnier, LoicYoon, Won-SupLentacker, IneYoshida, Go J.Lenzo, NatYoshida, HiroshiLeonardo, CostantinoYoshimoto, Francis K.Leonetti, CarloYoshimoto, YuyaLeonetti, CarloYoshino, HironoriLeong, Hon SingYoshioka, Ken-ichiLeporatti, StefanoYoshizaki, TomokazuLeporatti, StefanoYotsuyanagi, HiroshiLerario, Antonio M.You, XiaonaLeroux, DenisYou, ZongbingLeroy, KarenYoung, ArabellaLesueur, PaulYoung, JoanneLetizia, ClaudioYoung, TravisLeung, Ada W.Y.Yousefi, BardiaLeung, DilysYu, Cheng-ChiaLeung, DuncanYu, Cheng-PingLeung, IvanhoeYu, DiLeung, JustinYu, Kenneth H.Leung, Tin-ChungYu, WenboLevallet, GuénaëlleYu, Yan PingLevantini, ElenaYuan, Shyng Shiou F.Levi Sandri, Giovanni BattistaYuan, Shyng-Shiou F.Levy, Arkene S.A.Yuan, ZhenLevy, StevenYuan, ZhihongLewis, AnnabelleYudushkin, IvanLewis, Aurélia E.Yung, Hong-WaLewis, Stephen M.Yura, YoshiakiLi, ChiYvan-Charvet, LaurentLi, Chien-FengZaccara, SaraLi, DeminZadik, YehudaLi, FengzhiZaffaroni, NadiaLi, HaochengZaharoff, David A.Li, HaoxunZahid, MalihaLi, Hsing-HuiZaika, AlexanderLi, HuaZaiss, DietmarLi, HuiquanZakrevsky, PaulLi, Jian-JianZakrocka, IzabelaLi, JunZakrzewska, MalgorzataLi, JunjieZaman, AubhishekLi, LiZampese, EnricoLi, LianboZampieri, MattiaLi, Mei-LingZangemeister-Wittke, UweLi, MinZani, Bianca MariaLi, Shau-HsuanZaniboni, A.Li, Shengwen CalvinZannetti, AntonellaLi, ShurenZannetti, AntonellaLi, WeiZanzotto, Fabio MassimoLi, XiaohongZapico, Sara C.Li, XiaotaoZaporozhchenko, Ivan A.Li, YingZaretsky, Jesse M.Li, YunfeiZarobkiewicz, MichałLi, YvonneZavattaro, ElisaLi, ZheZavyalova, Elena G.Liagre, BertrandZdrowowicz, MagdalenaLian, Christine G.Zebisch, ArminLiang, Wei-ZheZehbe, IngeborgLiao, PengZeilinger, CarstenLiao, Yi-JenZeimet, Alain G.Libra, MassimoZeinomar, NurLibra, MassimoZeisel, Mirjam B.Liebman, MichaelZelionka, JacekLièvre, AstridZembutsu, HitoshiLignier, BaptisteZeng, BijunLim, BoraZeng, HaishanLim, BoraZeni, OlgaLim, Megan S.Zerdes, IoannisLim, StephanieZhai, KuiLim, Sung-JigZhang, ChiLimagne, EmericZhang, Daniel XinLin, AbrahamZhang, DianbaoLin, Been-RenZhang, ErshuaiLin, Chang-ShenZhang, FanLin, Chang-ShenZhang, JiLin, Chia-HuaZhang, JunLin, Chien-MinZhang, JunranLin, Chien-MinZhang, LeiLin, Chi-HungZhang, LiyingLin, Hai-ShuZhang, LuLin, Hsiu-MeiZhang, QiongLin, Huey-JenZhang, QiuyangLin, Kuo-ShyanZhang, RugangLin, Liang-InZhang, Shaun XiaoliuLin, Mei-HsiangZhang, WeicaiLin, ShengdaZhang, WeijieLin, Shi-MingZhang, WenyuLin, Song-SheiZhang, XiangLin, WeitaoZhang, XuexinLin, Y.Zhang, YanbinLin, YaoZhang, YanfeiLin, Ya-wenZhang, YumiaoLin, Ya-WenZhao, BailinLin, Yuan FengZhao, ChaoLinder, BenediktZhao, GexinLinder, StigZhao, HuiLindsay, HowardZhao, NingningLing, ChenZhao, QingnanLinnebacher, MichaelZhao, ShayingLiobikas, JuliusZhao, WeijiangLipkowitz, StanleyZhao, WenxiuLipton, AllanZhao, XiaoaiLisanti, Michael P.Zhao, YangbingLisowska, KatarzynaZheng, HongLita, AdrianZheng, Yao-RongLittle, Andrew C.Zhou, ChunxiaoLittle, JulianZhou, DawangLiu, BinZhou, GangLiu, CatherineZhou, Heshan SamLiu, Cheuk LunZhou, JiaLiu, Chi-MingZhou, JiajingLiu, Chun-YuZhou, JingyingLiu, David Xiao-MingZhou, LanLiu, DelongZhou, LinLiLiu, DongfangZhou, MinLiu, DongxuZhou, QingyuLiu, HongYanZhou, ShengmeiLiu, Jui-MingZhou, Stephanie QingyuLiu, LiZhou, WenchaoLiu, QunZhou, Yi-hongLiu, SiqiZhou, YiqunLiu, Wei-HsiuZhou, ZhijunLiu, XiangleiZhou, ZhixiongLiu, XiyongZhu, FanxiuLiu, YangZhu, HuaLiu, Yen-NienZhu, JunLiu, Yi-ChangZhu, ZheLiu, YingjunZhu, ZiwenLiu, YongqingZimpfer, AnnetteLiu, YuZiółkowski, PiotrLiu, Yu-PengZoidl, GeorgLiu, ZhongweiZölzer, FriedoLiu-Smith, FengZong, Wei-XingLizier, MichelaZoppoli, GabrieleLladser, AlvaroZoratti, MarioLleo De Nalda, AnaZordoky, BeshayLluis, FredericZuellig, RichardLo Muzio, LorenzoZuin, MatteoLo Sardo, FedericaZuo, ZhiliLo, Jeng-Fanzur Hausen, AxelLo, Leu-WeiZustovich, FableLo, SimonZwacka, RalfLo, U. Ging


